# Recent Advances in Lipid Nanoparticle-Mediated Respiratory and Gastrointestinal Mucosal Delivery of Nucleic Acids

**DOI:** 10.3390/bioengineering13080884

**Published:** 2026-07-31

**Authors:** Zefan Liu, Jiaqi Fu, Nan Mo, Juan Yang, Shenao Yan, Jing Hu, Minglu Zhou, Lian Li, Yucheng Xiang

**Affiliations:** 1 First People‘s Hospital of Shuangliu District (West China Airport Hospital of Sichuan University), Chengdu 610093, China; 2 Key Laboratory of Drug-Targeting and Drug Delivery System of the Education Ministry and Sichuan Province, Sichuan Engineering Laboratory for Plant-Sourced Drug, West China School of Pharmacy, Sichuan University, Chengdu 610041, China; 3 Department of Pharmacy, West China Hospital, Sichuan University, Chengdu 610041, China; 4 Sichuan Higher Education Institute Key Laboratory of Structure-Specific Small Molecule Drugs, Institute of Materia Medica, School of Pharmacy, Chengdu Medical College, Chengdu 610500, China

**Keywords:** nucleic acids, lipid nanoparticles (LNPs), mucosal barrier, drug delivery

## Abstract

The clinical translation of nucleic acids is severely hindered by multiple delivery barriers, such as enzymatic degradation, poor cellular uptake, endosomal entrapment, and rapid systemic clearance. Despite the remarkable therapeutic potential of these agents, conventional delivery systems often fail to address these challenges. Lipid nanoparticles (LNPs) have emerged as a versatile platform to overcome these obstacles, offering tunable physicochemical properties, high encapsulation efficiency, and pH-responsive endosomal escape. This review summarizes recent advances in LNP-based respiratory and gastrointestinal mucosal delivery of nucleic acids, with emphasis on formulation strategies for overcoming mucus and epithelial barriers. To overcome mucosal barriers, LNP studies have shown that keeping particle size below the local mucus mesh size (~100 nm), tuning surface charge toward near-neutrality via pH-responsive ionizable lipids, and maintaining a neutral, deformable, moderately PEGylated surface during the mucin transport stage can increase transmucosal diffusivity several-fold over conventional cationic LNPs. We further discuss current limitations and propose future directions, emphasizing the need for the integration of the pathological and physiological characteristics of specific mucosa with artificial intelligence (AI) platforms to develop intelligent and personalized delivery platforms with “spatiotemporal adaptive” capabilities.

## 1. Introduction

With the rapid development of molecular biology and biotechnology, biological macromolecules have become one of the most important areas in modern drug research and development. Biological macromolecules primarily include nucleic acid-based therapeutics (e.g., mRNA, siRNA, and gene editing systems) [1,2,3,4,5,6,7], protein and peptide drugs (e.g., insulin and glucagon-like peptide-1 analogues), as well as monoclonal antibodies [8,9,10,11,12]. In contrast to traditional small-molecule drugs, these agents exhibit superior target specificity and biological selectivity. Current biological macromolecules have demonstrated considerable advantages in the treatment of major diseases such as malignancies, metabolic disorders, and hereditary conditions [13,14,15,16,17,18,19,20,21]. Notably, the successful development and widespread application of mRNA vaccines in recent years further indicated that nucleic acid-based therapeutics have transitioned from experimental research to clinical translation and industrial-scale implementation, thereby propelling the rapid advancement of biologic macromolecular drugs [22,23].

Despite these advances, there are still substantial delivery-related challenges in the clinical translation of nucleic acids. Nucleic acid therapeutics are susceptible to enzymatic degradation and face transmembrane obstacles due to their polyanionic and highly hydrophilic nature. Even upon successful cellular entry, mRNA or siRNA molecules are often trapped within endosomes, creating a significant bottleneck for functional cytosolic delivery [24,25]. Furthermore, during systemic circulation, these therapeutic agents are subject to rapid clearance by the reticuloendothelial system (RES) and may undergo nonspecific binding with plasma proteins, thereby significantly reducing their bioavailability. Even if successful cellular internalization is achieved, a critical bottleneck remains in overcoming endosomal entrapment; failure to escape endosomal compartments results in lysosomal degradation, ultimately causing the loss of pharmacological efficacy [26].

The history of lipid-based nanocarriers can be traced back to the pioneering work of Bangham, who first characterized the self-assembly of phospholipids into closed bilayer vesicles, thereby establishing the fundamental physicochemical principles underlying lipid bilayer organization and its potential for encapsulating aqueous cargo [27,28,29,30,31,32]. Recent advancements, such as those reviewed in studies on nanostructured lipid carriers (NLC) and solid lipid nanoparticles (SLN) for applications like nasal delivery, further highlight their potential in targeted drug transport [33].

Building on these early insights, lipid nanoparticles (LNPs), a rapidly advancing nanocarrier platform, have emerged as an effective strategy to address the delivery challenges of biological macromolecules. Typically composed of ionizable lipids, cholesterol, helper phospholipids, and PEGylated lipids, LNPs can self-assemble into stable nanostructures. Nucleic acids can be efficiently encapsulated into LNPs, avoiding enzymatic degradation or immune clearance. Moreover, their surface properties and composition can be precisely modulated to prolong circulation time and optimize biodistribution. The ionizable lipids further enable endosomal escape via protonation in the acidic endosomal environment, facilitating efficient cytosolic delivery [34,35,36]. Owing to these advantages, LNPs have achieved clinical success with nucleic acid therapeutics and are increasingly being explored for protein and peptide delivery, holding significant translational promise.

This review systematically summarizes the fundamental properties of nucleic acids and their associated delivery barriers. In this review, the related literature in medical research databases from 2018 to 2026, including PubMed, Scopus, Web of Science, and ScienceDirect, was analyzed. Keywords such as LNP, mucosa, and terms related to drug delivery systems were analyzed. High-impact studies that represent mainstream delivery technologies and offer in-depth analysis of the key bottlenecks were selected as the core literature. The review focuses on recent advances in lipid nanoparticle (LNP)-mediated delivery of nucleic acids and further analyzes the influence of LNP formulation components on their physicochemical properties and delivery efficiency.

## 2. Overview and Research Status of Nucleic Acid Drugs

Biomacromolecular drugs refer to drugs obtained from microorganisms, cells, or biological tissues through biotechnologies such as genetic engineering, cell engineering, protein engineering, and fermentation engineering. They can be used to treat major diseases such as tumors, cardiovascular and cerebrovascular diseases, and immune diseases [26,37,38].

Nucleic acid drugs (NADs) are a class of gene therapy drugs based on DNA, RNA, or synthetic oligonucleotide analogs. NADs function directly at the genetic level and can be designed to target previously “undruggable” targets, making them suitable for genetic diseases, tumors, infectious diseases, and other conditions [21,39,40,41]. Based on their mechanism of action, NADs can be roughly classified into three categories: targeting nucleic acids, targeting proteins, and expressing proteins [42,43]. Table 1 lists the technical principles and representative marketed or investigational products for each category.

Although nucleic acid drugs can directly regulate gene expression and treat rare genetic diseases, they have several inherent limitations in in vivo application. For example, in the bloodstream, unmodified nucleic acids are rapidly degraded by nucleases (DNase/RNase) in the plasma and tissues, with a half-life of only a few minutes to tens of minutes. Regarding cellular uptake, nucleic acid molecules carry a dense negative charge and cannot passively diffuse through the negatively charged phospholipid bilayer of the cell membrane, resulting in extremely low cellular uptake efficiency [30,59,60]. Therefore, developing an efficient, specific, and safe delivery system remains the core challenge currently faced in the research and development of nucleic acid drugs.

## 3. LNPs and Delivery

Compared with traditional liposomes and high-molecular-weight polymers, lipid nanoparticles (LNPs) not only have a better encapsulation rate and excellent stability in blood circulation, but also significantly enhance the cytoplasmic utilization rate of NADs through their unique intracellular escape mechanism [61]. From the limitations of retroviral delivery to the successful support of global mRNA COVID-19 vaccines in clinical applications, LNPs have become an indispensable bridge connecting NADs and clinical translation [62].

### 3.1. LNP Overview

**Figure 1:**
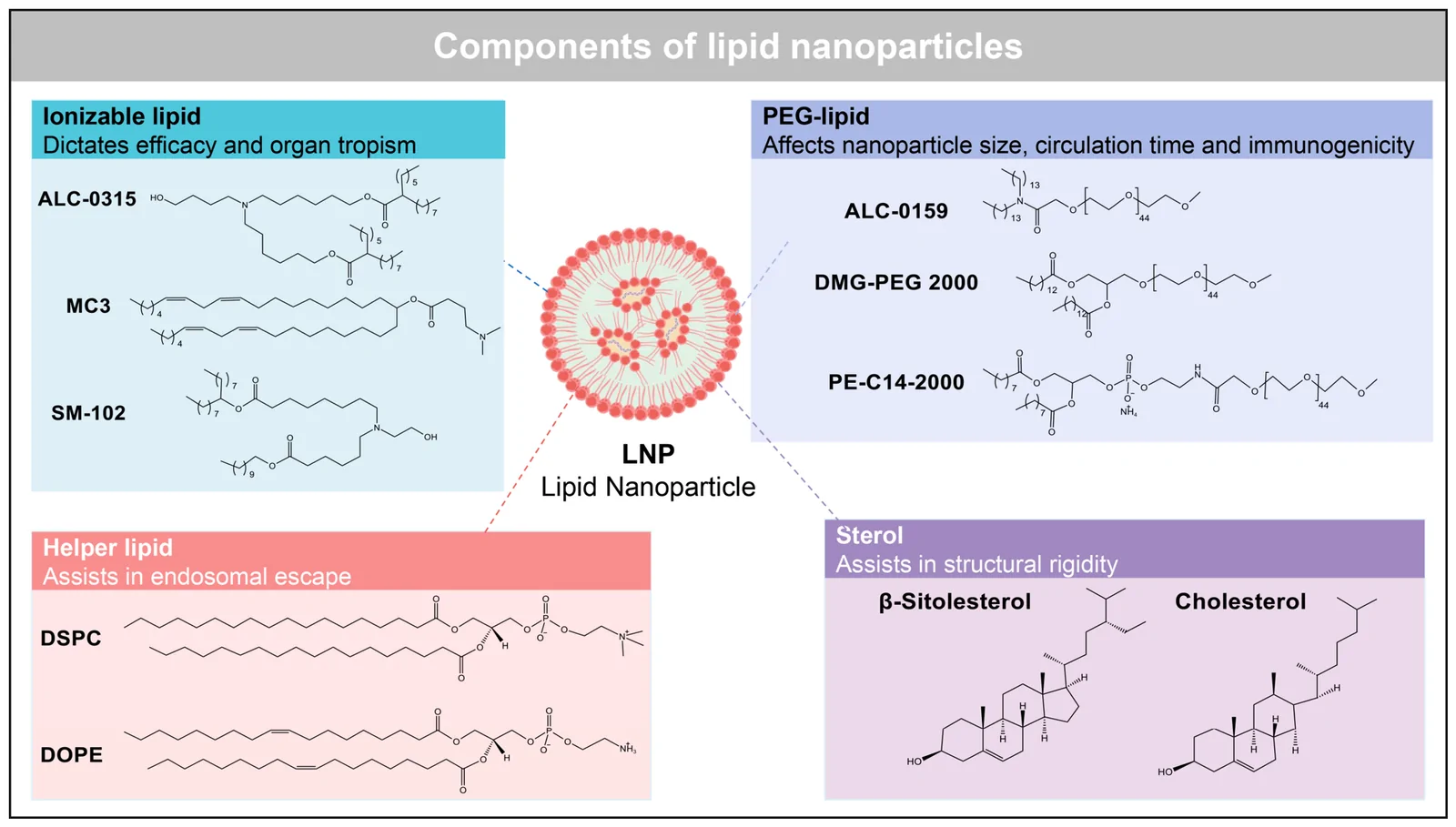
Components of lipid nanoparticles.

Unlike the strictly aqueous-core structure of traditional liposomes, LNPs exhibit diverse internal morphologies, ranging from solid-like or intermediate cores to classic liposome-like bilayer structures, depending heavily on their composition. LNPs are typically formed by ionizable cationic lipids, helper lipids, cholesterol, and PEGylated lipids in nanoscale proportions (Figure 1).

Ionizable cationic lipids are the core functional components of LNPs. At physiological pH, they remain electrically neutral, effectively reducing non-specific interactions with blood components and lowering systemic toxicity. In the acidic environment of endosomes (pH < 6.0), they become protonated and positively charged, driving subsequent membrane fusion and endosomal escape.

Helper lipids are often selected from distearoylphosphatidylcholine (DSPC) or dioleoyl phosphatidylethanolamine (DOPE). Helper lipids are mainly distributed in the outer shell of LNPs to stabilize the encapsulation structure.

Cholesterol can regulate the fluidity and rigidity of the LNP shell, limit the lateral diffusion of lipid molecules, and enhance particle stability.

PEGylated lipids prevent the aggregation of LNPs through the steric hindrance effect of PEG [63,64,65].

The formation of LNP is a multi-component self-assembly process that is jointly regulated by lipid composition, pH, mixing kinetics, and solvent environment. Generally, ionizable lipids, after being protonated under acidic conditions, form electrostatic interactions with negatively charged nucleic acid drugs. Meanwhile, lipid molecules drive the self-assembly of lipids into nanoparticles through hydrophobic effects, van der Waals forces, and other interactions [66].

Modern industry and laboratories commonly use microfluidic technology to prepare LNPs. Through microfluidic chips, the prepared LNPs have a narrow particle size distribution and highly consistent encapsulation efficiency [67,68].

### 3.2. Research Progress in the Application of LNPs

**Figure 2:**
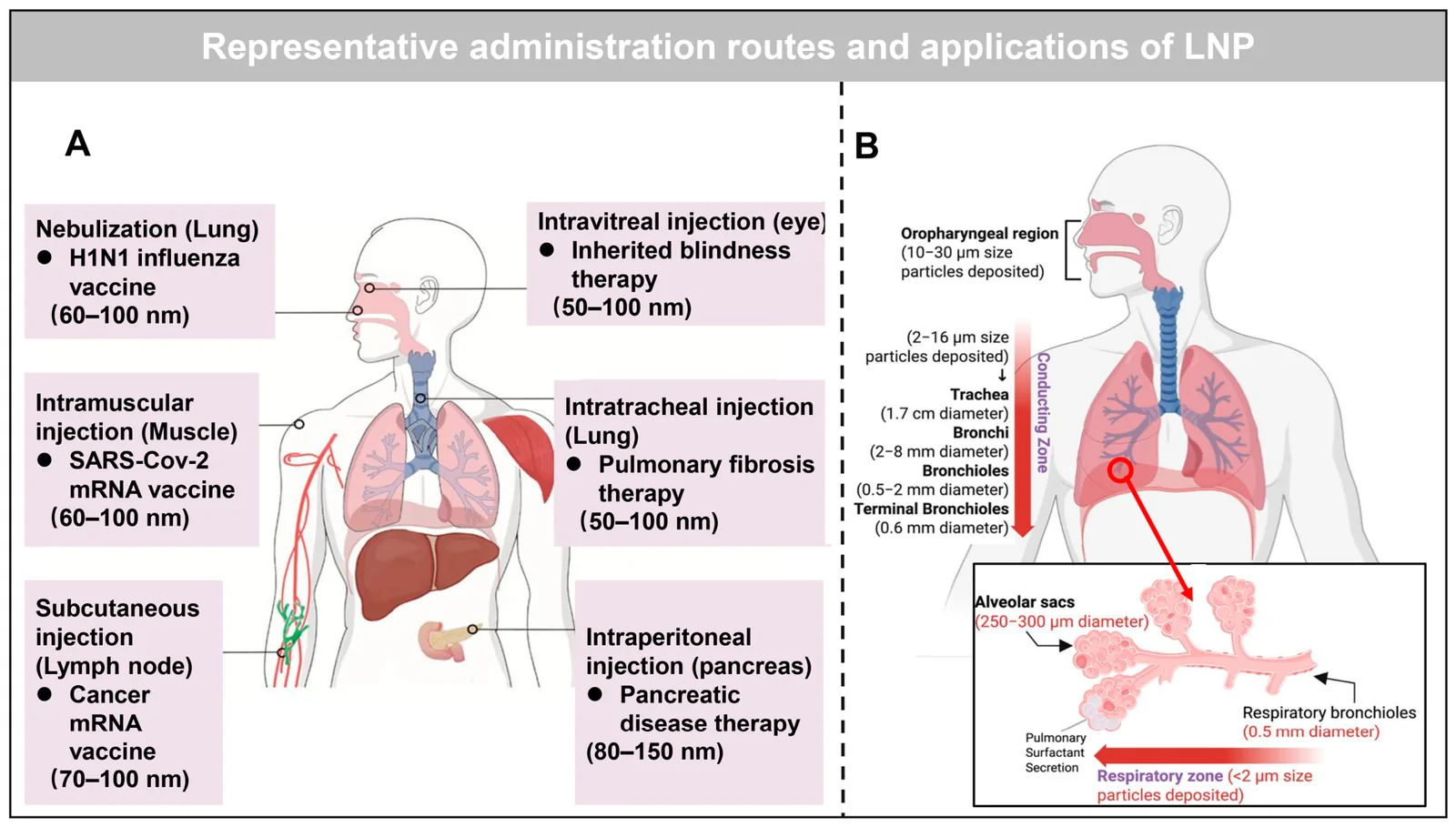
Representative administration routes and applications of LNP. (**A**) Reprinted with permission from [70]. (**B**) Reprinted with permission from [78].

LNPs are currently the most widely used non-viral nucleic acid delivery vectors in commercial vaccines and gene therapy products (Figure 2). However, following systemic administration, they tend to accumulate preferentially in the liver, limiting efficacy for extrahepatic targets. Recent breakthroughs in altering formulation composition or chemically modifying ionizable lipids have enabled organ-selective targeting beyond the liver. Concurrent mucosal administration has emerged as a non-invasive alternative, although LNP designs that specifically overcome the mucosal barrier are required. The following sections therefore review the latest progress in LNP delivery strategies.

#### 3.2.1. Injection Administration

Injection administration is currently the most mature and widely used administration method for LNPs in clinical translation [69,70]. LNPs can effectively protect nucleic acids from degradation by complex nucleases in the blood and achieve efficient intracellular release after overcoming multiple biological barriers. With an in-depth understanding of the structure-activity relationship of lipid molecules and the introduction of advanced engineering platforms, the design of injectable LNPs has transitioned from the initial stage of “endogenous passive enrichment” to a new stage of “programmed precise targeting” and “fine organ selection” [71]. Table 2 summarizes the organ-specific targeting strategies.

**Table 2:** Summary of organ-specific LNP targeting strategies.

Organ	Targeting Strategy	Key Modifications/Lipids	Representative Applications	Significance
Liver	Endogenous ApoE adsorption	Conventional LNPs	Patisiran (Onpattro^®^) [72]	Clinical benchmark
	LDLR-mediated	Oxidized cholesterol [61]		
Spleen	The selective organ targeting SORT strategy	Anionic lipid tuning	Spleen-specific mRNA delivery [73]	Overcomes liver dominance for immune organs
	AI-guided design	P1 LNP (tritail conical ionizable lipid)	enhanced vaccine immunogenicity via IgM/FcμR [74]	
Bone/Bone Marrow	Bisphosphonate affinity	BP-modified ionizable lipids	Bone mineral anchoring [75]	Overcomes dense bone matrix barrier
	antibody coupling	anti-CD117 antibody	hematopoietic stem cell targeting [76]	
Muscle (IM)	Specialized topological ionizable lipids	Iso-A11B5C1	High local expression [77]	Vaccine optimization and toxicity reduction

#### 3.2.2. Inhalation/Nasal Administration

The respiratory tract and lungs are the primary sites where various lethal infectious diseases, genetic disorders, and malignant tumors occur. The use of LNPs to deliver therapeutic mRNA or antibody-coding sequences has shown significant clinical potential in this field.

##### Aerosol and Formulation Factors for Inhaled LNPs

While the hydrodynamic diameter of nanoparticles is widely recognized as a determinant of cellular uptake and mucus penetration, the regional deposition of inhaled nano-formulations in the lung is critically governed by their aerosol properties. This performance is shaped by multiple interrelated factors, including nebulizer class, aerosol output rate, aerodynamic diameter, formulation concentration, viscosity, osmolality, and the structural integrity of nanoparticles after nebulization. Jet nebulizers, though compatible with a broad range of nanocarriers, often produce heterogeneous droplet sizes, generate significant noise, and leave high residual volumes, leading to variable drug output and potential dose wastage [79]. Ultrasonic nebulizers offer faster delivery and lower noise but are limited by heat generation, which can denature thermolabile biomolecules, alter formulation viscosity and concentration through solvent evaporation, and promote particle agglomeration [80]. In contrast, mesh nebulizers produce a slow-moving aerosol with a narrow droplet size distribution, low residual volume (0.1–0.5 mL) and high respirable fraction, without appreciable heating, making them particularly suitable for protein-based and other heat-sensitive nano-formulations [78]. Furthermore, the aerodynamic diameter, which is ideally in the 1–5 μm range for deep lung deposition, determines the deposition mechanism (inertial impaction, sedimentation, or diffusion) and is influenced by both the initial droplet size and any post-nebulization changes in nanoparticle size or aggregation state [78]. Formulation parameters such as concentration, viscosity, and osmolality can affect droplet generation efficiency and aerosol output, while also influencing the shear stress experienced by nanoparticles during atomization. High viscosity or hyperosmolarity may impair nebulization performance or compromise colloidal stability [79]. Crucially, post-nebulization integrity, which is assessed by changes in particle size, polydispersity, drug entrapment, or release profile, is often overlooked. However, it directly determines the therapeutic efficacy of the delivered nanomedicine. Overall, a thorough characterization of these aerosol properties, rather than reliance solely on hydrodynamic size, is essential for predicting regional deposition and for rational device-formulation matching in inhaled nanomedicine.

##### Current Research

As of July 2026, clinical-stage inhalable LNP formulations mainly focus on cystic fibrosis. MRT5005 (ClinicalTrials.gov identifier: NCT03375047) evaluated single-dose (8–24 mg) and multiple-dose (up to 20 mg per week or 4 mg per day) regimens in phase I/II trials. However, MRT5005 did not consistently improve lung function and was accompanied by notable systemic adverse reactions such as fever. This study has been completed, and the Cystic Fibrosis Foundation has classified it as terminated, with no ongoing research progress [81]. VX-522 (ClinicalTrials.gov identifier: NCT05668741) once entered the multi-dose escalation stage. However, VX-522 has currently been terminated due to tolerability issues. Clinical progress indicates that airway and local immune responses are the key bottlenecks for the current inhalable LNP to move toward clinical application [82].

Research by Bai et al. indicates that optimized inhalable LNPs can mediate efficient pulmonary antibody therapy to alleviate fibrotic lesions [83]. Xu et al. employed lactoferrin-functionalized LNPs for intranasal delivery of co-encapsulated α-mangostin and BACE1 siRNA, achieving synergistic therapy for Alzheimer’s disease [84]. Inhalation administration is an efficient non-invasive delivery strategy that enables LNPs to directly act on the respiratory mucosa and epithelial surface, establishing a high therapeutic concentration at the lesion site, while avoiding the first-pass effect and systemic toxicity associated with systemic administration [85]. However, the complex mucus barrier in the airways, the clearance mechanism of alveolar macrophages, and the physical shear forces during nebulization pose significant challenges to the stability of inhaled LNPs [86,87].

**Figure 3:**
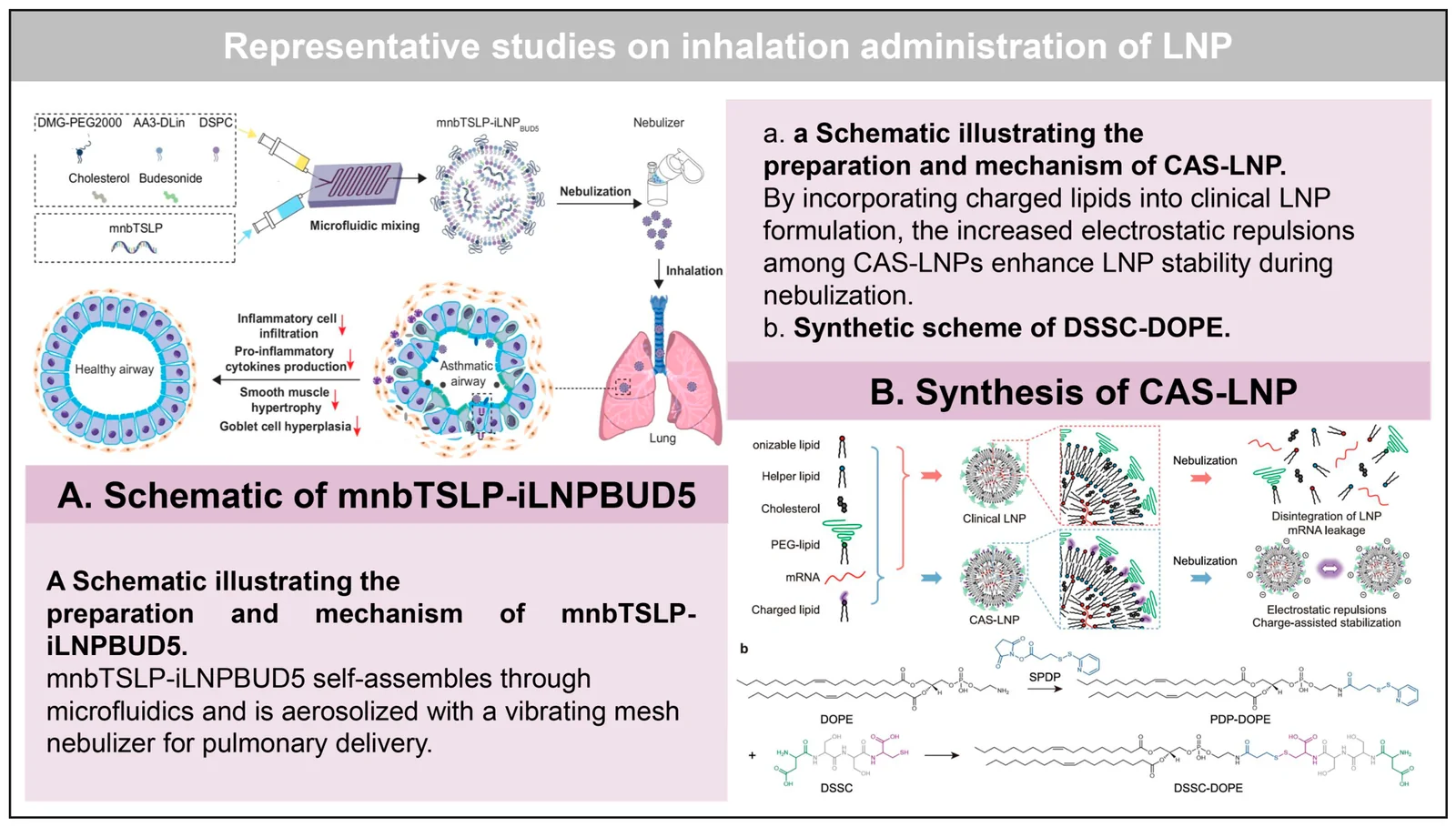
Representative studies on inhalation administration of LNP. (**A**) Reprinted with permission from [92]. (**B**) Reprinted with permission from [90].

Commonly used clinical nebulization devices generate strong mechanical shear forces and thermal effects during operation, which can easily lead to the disintegration, aggregation, or premature degradation of RNA in traditional LNPs, thereby significantly reducing their transfection efficiency. To enhance their physical stability against shear forces, researchers have fine-tuned LNP formulations, for example by appropriately increasing the molar ratio of PEG-lipid or introducing high-molecular-weight hydrophilic branched polymer excipients into the formulation to build a strong steric barrier on the nanoparticle surface [86]. The research conducted by Allen Y. Jiang et al. incorporated the high-molecular-weight hydrophilic branched polymer excipient bPEG20K into the LNP formulation, demonstrating that even under the intense mechanical shear forces and local high temperatures generated during the aerosolization process, a powerful spatial steric hindrance barrier could effectively maintain the particle size of LNPs [88]. In parallel, Ke Huang et al. further improved the structural tolerance of LNPs under aerosolization shear forces by introducing a new type of degradable glycerol esterified ionizable lipid. This modification not only effectively inhibited particle aggregation during the aerosolization process but also enhanced the ability of particles to penetrate the respiratory mucus layer, significantly improving the expression efficiency of mRNA in the deep regions of the lungs [89]. Liu Shuai et al. employed the charge-assisted stabilization (CAS) strategy and incorporated the DSSC-DOPE peptide-lipid conjugate into the formulation, resulting in the generation of negative charges (approximately −10 mV) on the surface of the LNP. The resulting CAS-LNP maintained approximately 59% integrity during the atomization process, which was significantly higher than the 17% retention rate of the conventional LNP [90]. Based on this, Cheng et al. utilized the mucolytic agent N-acetylcysteine (NAC) to restore cellular uptake efficiency. After NAC was deposited in the lungs, it could remove the DSSC peptide from the surface of CAS-LNP and exert a mucolytic effect [91]. The structural stability of inhaled LNPs also laid the foundation for their multi-component combined treatment (Figure 3). Jia Huang’s research innovatively encapsulated the small molecule glucocorticoid budesonide and the anti-TSLP nanobody mRNA together within a shear-resistant inhaled LNP, achieving efficient local synergistic treatment for asthma [92].

Furthermore, significant progress has been made in the research of inhaled mRNA-LNP vaccines. Shuai Liu et al. developed an improved LNP through a charge-assisted stabilization (CAS) strategy. They introduced the negatively charged peptide-lipid conjugate DSSC-DOPE into the SM-102-based formulation to enhance the colloidal stability of the LNP during the aerosolization process. Multi-species validation was conducted in mice, dogs, and pigs. In ex vivo imaging 24 h after administration in mice, CAS-LNP mainly targeted the lungs, while no detectable hepatic signal was detected under the reported imaging conditions, contrasting sharply with systemic injection. These LNPs mainly transfected dendritic cells (>60%), effectively eliciting vaccine protective effects against the SARS-CoV-2 Omicron variant and lung metastatic cancer [90]. In Cheng et al., in the B16F10-OVA lung metastasis and subcutaneous tumor model, the NAC + CAS-LNP vaccine achieved complete cure in 6 out of 8 mice and tumor regression in the subcutaneous tumors. Moreover, it provided long-term protection after re-infection. The systemic anti-tumor effect of the vaccine was comparable to that of the mRNA vaccine administered intramuscularly [91]. Hu et al. developed an inhalable LNP that codelivers an anti-DDR1 single-chain antibody mRNA (mscFv) with a PD-L1-targeting siRNA. Inhalation of mscFv/siPD-L1@LNP significantly promoted the infiltration of CD8^+^ T cells and NK/NKT cells, achieved tumor regression and prolonged overall survival in LLC lung cancer and 4T1 lung metastasis models [93]. Gabriela Baldeon Vaca et al. evaluated two LNP formulations, mRNA-LNP 1 and mRNA-LNP 2, in Syrian golden hamsters. mRNA-LNP 1 was based on the clinical formulation of Moderna, while mRNA-LNP 2 additionally contained the cationic lipid DOTAP. A 25 μg dose administered by nasal inhalation induced S-specific serum IgG comparable to that induced by 0.4–1 μg intramuscular injection. After viral challenge, the 25 μg nasal vaccination group showed a relative reduction in viral load in the lungs and nasal concha on the third day and compared to the 22.6% N protein^+^ cells in the control group, the high-dose group significantly reduced the percentage of N protein^+^ cells in the lungs on the 14th day, with more virus clearance by day 14. Crucially, nasal vaccination stimulated S-specific IgG and IgA serum antibodies, with the IgA response being crucial for respiratory defense [94]. Mai N. Vu et al. in C57BL/6 and Ai14 reporter gene mice, deeply analyzed the LNP cell targeting mechanism and compared the effects of intramuscular and nasal administration. Although LNPs containing DOTAP showed strong transfection in the lungs, their immunogenicity was extremely poor, indicating that antigen expression on respiratory epithelial cells and immune cells was insufficient to drive effective mucosal immunity; conversely, LNPs without DOTAP achieved better immune balance [95]. In summary, respiratory delivery successfully bypasses hepatic clearance and, more importantly, triggers localized mucosal IgA responses to establish front-line defense, presenting a distinct immunological profile from conventional intramuscular injection despite its lower efficiency in driving systemic IgG.

#### 3.2.3. Oral Administration

Oral administration has high patient compliance and convenience, making it an ideal route for long-term treatment of chronic diseases and digestive system disorders (Table 3). However, the gastrointestinal environment constitutes a harsh biological barrier. The extremely acidic pH environment in gastric juice directly destabilizes the structural integrity of LNPs. Pancreatic lipase and bile salts in the small intestine easily break down and dissolve the lipid matrix [96]. In addition, the dense mucus layer and tight junctions on the intestinal epithelial surface severely impede the trans-epithelial transport of nanoparticles [70,97,98].

To overcome the aforementioned multiple physiological barriers, researchers have combined LNP technology with advanced macro/micro biomaterials science to develop environment-responsive multi-level composite delivery systems. The core strategy is to further micro-encapsulate LNPs within high-molecular-weight polymer materials that are resistant to gastric acid and have controllable release in the intestine [60,99]. Interestingly, ionizable lipids are crucial for the endosomal escape of LNPs. Ionizable lipids can effectively promote the release of mRNA from endosomes into the cytoplasm, while relying solely on permanent cationic lipids is likely to cause endosomal entrapment, significantly reducing the delivery efficiency [100].

Based on this, Kanika Suri et al. significantly improved the structural stability and nucleic acid encapsulation efficiency of LNPs in biocompatible media by systematically introducing permanent cationic lipids into the classic ionized lipids, while retaining efficient endosomal escape ability [101]. The OrD LNP platform successfully achieved siRNA-mediated gene silencing and mRNA-driven protein expression in mouse models. Po-Kai Luo et al. encapsulated mRNA with β-glucan pre-complexed into pH-responsive LNPs, enabling the complex to achieve effective protection in the harsh environment of the gastrointestinal tract and triggering release in the intestinal immune cell microenvironment, thereby promoting the in situ expression of tumor antigens and systemic anti-tumor immune activation [102]. Md. Anamul Haque et al. developed a pH-sensitive polymer-coated LNP system for oral nucleic acid delivery, named Eu-LNPs. Eudragit^®^ S 100 is insoluble in the acidic environment of the stomach but dissolves in the neutral to alkaline (pH approximately 7) intestinal environment. Therefore, Eu-LNPs can effectively protect mRNA-LNP under the extremely acidic and enzyme-rich barrier of the stomach. When entering the neutral/alkaline environment of the intestine, Eudragit^®^ S 100 dissolves and releases the LNP, restoring its efficient transfection and expression [103].

In addition, some nano-drug delivery systems have also adopted the characteristics of LNPs for their design. Xiangang Huang et al. developed RNACap using the cationic lipid-like material G0-C14 to provide positive charge and a proton sponge effect to facilitate endosomal escape. RNACap shields mRNA from gastric acid via a pH-sensitive coating and releases its contents into the intestine. Oral RNACap increased local IL-10 expression and reduced disease markers in a rat model of experimental colitis. Stability and release behavior of RNACap were further confirmed in a porcine model, providing preliminary evidence that capsule-based protection strategies may be scalable beyond rodents [104]. Chae Won Cho et al. developed a pH-responsive bilayer-coated macrophage-derived exosome (DCEVs) for the treatment of colitis. The DCEVs were coated with the cationic lipid DOTAP to achieve charge reversal and endosomal escape. Then the DCEVs were covered with sodium alginate (SA) + 5-ASA to enhance gastrointestinal stability and colonic-targeted release [105].

Collectively, oral delivery of nucleic-acid-loaded LNPs is currently achieved through pH-protective encapsulation. However, development remains largely preclinical.

**Table 3:** Comparison of the respiratory and gastrointestinal mucosal routes for LNP delivery systems.

LNP	Formulation	Cargo	Dose	Animal Model	Administration Device	Quantitative Endpoint	**Duration**	**Principal Limitation**
mnbTSLP-iiLNP_BUD5_ (ASCEND) [92]	AA3-DLin:DSPC:Cholesterol:Budesonide:DMG-PEG 2000 = 60:20:14:5:1	mRNA	30 μg mnbTSLP per mouse	Mice	vibrating mesh nebulizer	A body weight loss of ≥20%	21–28 days	Species translational gap Long-term Safety
IR-117-17 LNPs [88]	C12-200: DOTAP:Chol:PEG 2000 = 35:28:34.5:2.5	mRNA	0.2 mg mRNA per mouse	Mice	vibrating mesh nebulizer	Ashcroft	24–72 h	Species translational gap High-dose aggregation Long-term Safety
iLNP_HP08_ [83]	AA3-DLin:DSPC:Cholesterol:DMG-PEG2000 = 60:20:19:1	mRNA	15 μg mRNA per mouse	Mice	vibrating mesh nebulizer	Ashcroft	28 days	Species translational gap Long-term Safety Scalable manufacturing challenge
si/pD-DAS-LNP (Asiaticoside modified) [106]	DLin-MC3-DMA:DOPE:DPPC:DMG-PEG2000:AS = 50:10:20:1.5:18.5	siRNA; pDNA	0.1 mg/kg siRNA/ pDNA	Mice	intratracheal microsprayer aerosolizer	Ashcroft	28 days	Species translational gap Long-term Safety
α-M/siB@L-Lf [84]	SM-102:DSPC:Cholesterol:DMG-PEG2000:DSPE-PEG2000-Mal = 42:13:43:1.5:0.5.	siRNA	0.5 mg/kg α-M/siB@L-Lf	Mice	intranasal	N/A	23 days	Species translational gap
OrD LNP [101]	C12-200:DOTMA:DSPC:Cholesterol:DMG-PEG = 30:20:10:38.5:1.5	siRNA	2 mg/kg OrD LNP	Mice	oral	efficacy to knock down endogenous HPRT gene	6 h	Long-term Safety
Eu-LNPs [103]	DLin-MC3-DMA:Cholesterol:DMG-PEG:DSPC = 50:38.5:10:1.5	mRNA	N/A	N/A	oral	N/A	N/A	No in vivo studies
CAS-LNP [90]	SM-102:DSPC:Cholesterol:DMG-PEG2000:DSSC-DOPE = 50:7.5:38.5:1.5:2.5	mRNAs	5 µg mRNA per mouse	Mice; Dogs/Pigs	oropharyngeal aspiration	Ashcroft	37 days	Model Limitations
βGlus/mRNA@LNPs [102]	SM-102:DSPC:cholesterol:DMG-PEG 2000 = 50:10:38.5:1.5	mRNA	20 μg βGlus/mOVA@LNPs	Mice	oral	tumor size reached 2000 mm^3^	27 days	Antitumor Effect Species translational gap

#### 3.2.4. Other Mucosal Routes

In addition to the major delivery routes discussed above, several other mucosal sites have also been explored as viable entry points. The oral cavity, eyes, and reproductive tract also possess extensive vascular networks and immune-active cells. Hana Esih et al. developed a dual-layer mucosal adhesion membrane (MAF) drug delivery system, which was delivered through the buccal mucosa. MAF successfully loaded DNA plasmids, viral vectors, and mRNA/LNP vaccines, inducing systemic IgG, antigen-specific cytotoxic T cell responses, and local mucosal IgA production, achieving protection against respiratory tract infections [107]. Ibe Van de Casteele et al. designed a self-amplifying sa-mRNA encapsulated LNP drug delivery system, delivered through vaginal mucosal spray, achieving local LNP uptake and sa-mRNA expression in pig models, verifying its potential for targeted prevention of sexually transmitted infections (STIs) [108]. Yukako Taketani et al. used LNP to encapsulate proline-modified short hairpin anti-ANGPTL2 RNA designed as ANGPTL2 Li-pshRNA, delivered topically through ocular mucosal eye drops, successfully inhibiting corneal ANGPTL2 mRNA expression and significantly reducing corneal neovascularization (CNV) in a mouse model of alkali burn [109].

Collectively, while the oral cavity, eyes, and reproductive tract are not the primary focus of current LNP delivery research, they represent promising applications worth continued investigation.

### 3.3. Strategies to Overcome Mucosal Barriers

LNPs must first traverse the mucus layer and subsequently be recognized and internalized by the underlying epithelium. These two stages impose partially conflicting surface requirements. Mucus penetration favors an inert, near-neutral, hydrophilic surface, whereas epithelial or endosomal engagement typically requires transient surface reactivation like charge conversion, PEG shedding, and membrane fusion [110]. The following sections address particle size, surface charge, hydrophilicity, and membrane fluidity as design parameters, and summarize the influence of each design parameter on the transmembrane behavior of LNPs.

#### 3.3.1. Particle Size Regulation

The mucus layer covering the mucosal surface is essentially a mesh-like structure formed by cross-linked mucin fibers, with mesh sizes typically ranging from 20 to 200 nm and showing high heterogeneity [111]. When the hydrodynamic particle size is larger than the mesh size of the mucus, the nanoparticles are physically trapped in the fiber network [112]. This size-sieving phenomenon was first characterized by chemically inert model nanoparticles. In a human airway mucus system, Schuster et al. showed that nanoparticles of 100 nm and 200 nm could freely diffuse through the mucus mesh, while 500 nm nanoparticles were trapped in the mucus [113].

Similarly, in LNPs, Tafech et al. reported that cystic fibrosis mucus exhibits markedly smaller pore sizes (60–200 nm) than healthy mucus (100–500 nm), and that mRNA-loaded LNPs (~42 nm) diffused roughly twice as fast as the larger RNP-loaded LNPs (~279 nm) in healthy porcine mucus [114]. The muco-penetrating iLLN-2 formulation for intranasal mRNA delivery (~100 nm) is situated within the reported mucus mesh range. Compared with conventional ALC-0315-based LNPs, iLLN-2 markedly enhanced nasal transfection [115].

Importantly, particle size also governs the downstream epithelial/cellular-uptake step. The optimal window for mucus penetration does not necessarily coincide with the optimal window for cellular internalization or antigen-presenting cell (APC) uptake. Tam A et al. reported that sub-100 nm LNPs preferentially penetrate mucus and transfect airway epithelial cells, whereas larger LNPs (200–500 nm) are more efficiently taken up by dendritic cells and favor systemic immune priming [116]. This size-dependent trade-off between mucus diffusivity and target-cell uptake indicates that particle size alone cannot be optimized in isolation. It must be selected according to the intended downstream target.

#### 3.3.2. Charge Regulation

Under physiological pH conditions, the mucus layer exhibits a strong negative charge, primarily due to the presence of highly glycosylated sialic acid residues and sulfate groups in the mucin backbone [117]. Therefore, the surface charge of LNPs directly determines whether they will electrostatically adhere to the mucus. In a bovine submaxillary mucin hydrogel system, Tafech et al. showed that reducing the net negative charge of the mucin network increased LNP-mRNA diffusivity roughly three-fold, and that increasing the ionic strength of the medium (0.01 mM to 160 mM) raised the diffusion coefficient from 0.15 to 0.60 μm^2^/s by Debye screening of LNP–mucin electrostatic attraction. Brownian dynamics simulations further confirmed that both electrostatic repulsion and electrostatic attraction impede LNP diffusion, with a near-neutral LNP–mucin interaction potential yielding maximal diffusivity [114]. It should be noted that this bovine submaxillary mucin hydrogel is a simplified in vitro reconstitution and may not fully recapitulate the native airway mucus in vivo.

**Figure 4:**
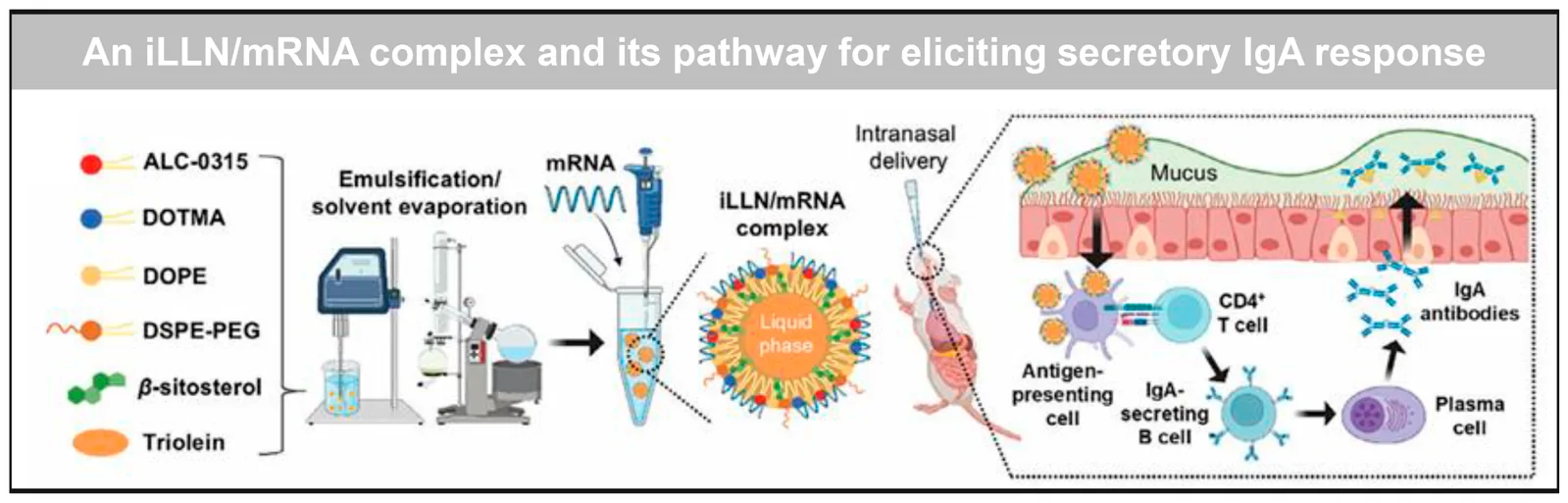
An iLLN/mRNA complex and its pathway for eliciting secretory IgA response [115]. Adjusting the ratios of ionizable and cationic lipids allows fine-tuning of the pKa of iLLNs to the range of nasal mucosal pH (5.5–6.5), thus facilitating mucus penetration via the formation of near-neutral, PEGylated muco-inert surfaces. A prime-boost intranasal immunization of iLLN-2/mRNA complexes can elicit a greater magnitude of SARS-CoV-2 spike-specific mucosal IgA and IgG response than ALC-LNP, without triggering any noticeable inflammatory reactions. Reprinted with permission from [115].

By varying the molar ratio of the ionizable lipid ALC-0315 and the cationic lipid DOTMA, the iLLN formulations achieved apparent pKa values ranging from 5.57 to 7.22 (Figure 4). The optimal formulation (iLLN-2, apparent pKa = 5.94) formed a near-neutral surface (zeta potential ≈ +4.3 mV as an mRNA complex) at nasal mucosal pH (~6). It exhibited an apparent mucus permeability coefficient roughly 2.7-fold higher than the strongly cationic formulation (cLLN, zeta potential ≈ +30.2 mV) [115]. Similarly, the pulmonary DAS-LNP formulation maintained a slightly negative zeta potential and showed minimal protein adsorption upon incubation with mucin solution [106].

**Figure 5:**
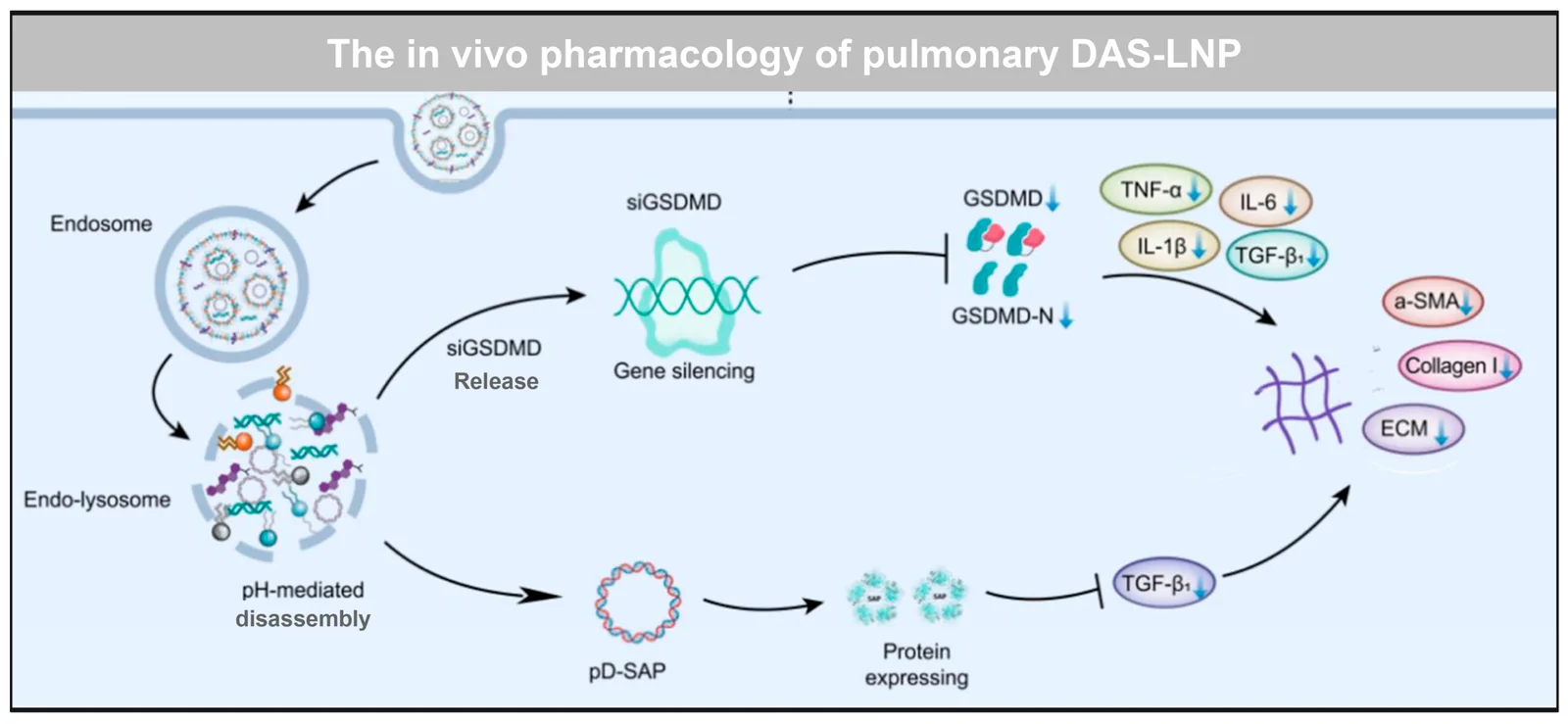
Schematic on the in vivo pharmacology of pulmonary DAS-LNP [106]. siGSDMD decreases GSDMD-mediated pyroptosis and the subsequent release of inflammatory mediators, while SAP expression restricts aberrant fibroblast activation and extracellular matrix deposition. Collectively, these effects are anticipated to alleviate the progression of idiopathic pulmonary fibrosis (IPF). Reprint with permission from [106].

Interestingly, surface charge is not only a static mucus-penetration parameter but also the principal mechanism by which LNPs reconcile mucus-inertness with subsequent epithelial and endosomal engagement, via pH-responsive charge conversion. For example, iLLN is built on an ionizable lipid. Its surface charge is inherently pH-dependent. The apparent pKa of iLLN-2 is 5.94, which is lower than the extracellular/mucosal pH value (~6–7.4). At this stage, the particles are close to being neutral. The pH range of endosomes from the early to the late stage is 5.5–6.5. At this time, the tertiary amine head group of iLLN-2 can recover cationic charge through protonation. At this point, iLLN-2 can fuse with endosomal phospholipids through electrostatic interactions. Maniyamgama et al. demonstrated that the iLLN-2/mRNA complex achieved 69.5% endosomal escape in A549 cells, while the traditional ALC-0315 LNP only had 35.8% [115]. A similar strategy was adopted in the lung DAS-LNP platform (Figure 5). Gu et al. replaced cholesterol with snowberry glycoside and DPPC to increase the apparent pKa to 6.30. This strategy improved the co-localization of nucleic acids with Lyso Tracker-positive compartments and enhanced lysosome escape 6 h after transfection [106]. These studies collectively indicate that a suitable ionizable lipid pKa is conducive to LNPs achieving mucin-inert transport and triggering endosomal escape.

Notably, the charge-based evidence summarized above characterizes electrostatic surface potential. Available data rely mainly on indirect proxies, including zeta potential measurements of both mucin and LNP surfaces across a range of pH values or others. Direct quantification of mucosal surface free energy, for instance, by contact-angle measurement or atomic force microscopy, remains absent from the current LNP-mucus interaction literature. We believe this represents an important direction for future characterization of the mechanism of LNP-mucosal interaction.

#### 3.3.3. Hydrophobicity and Hydrophilicity Regulation

The hydrophobicity and hydrophilicity of LNPs are another factor affecting their mucosal absorption. In the mucus layer covering the mucosal surface, the protein core of mucin fibers is interspersed with numerous hydrophobic domains, which readily non-specifically bind to other hydrophobic surfaces. Consequently, hydrophilic, PEGylated surfaces generally outperform hydrophobic ones in mucus transit. This principle was already evident in the polystyrene nanoparticles prepared by Lai et al. [118].

**Figure 6:**
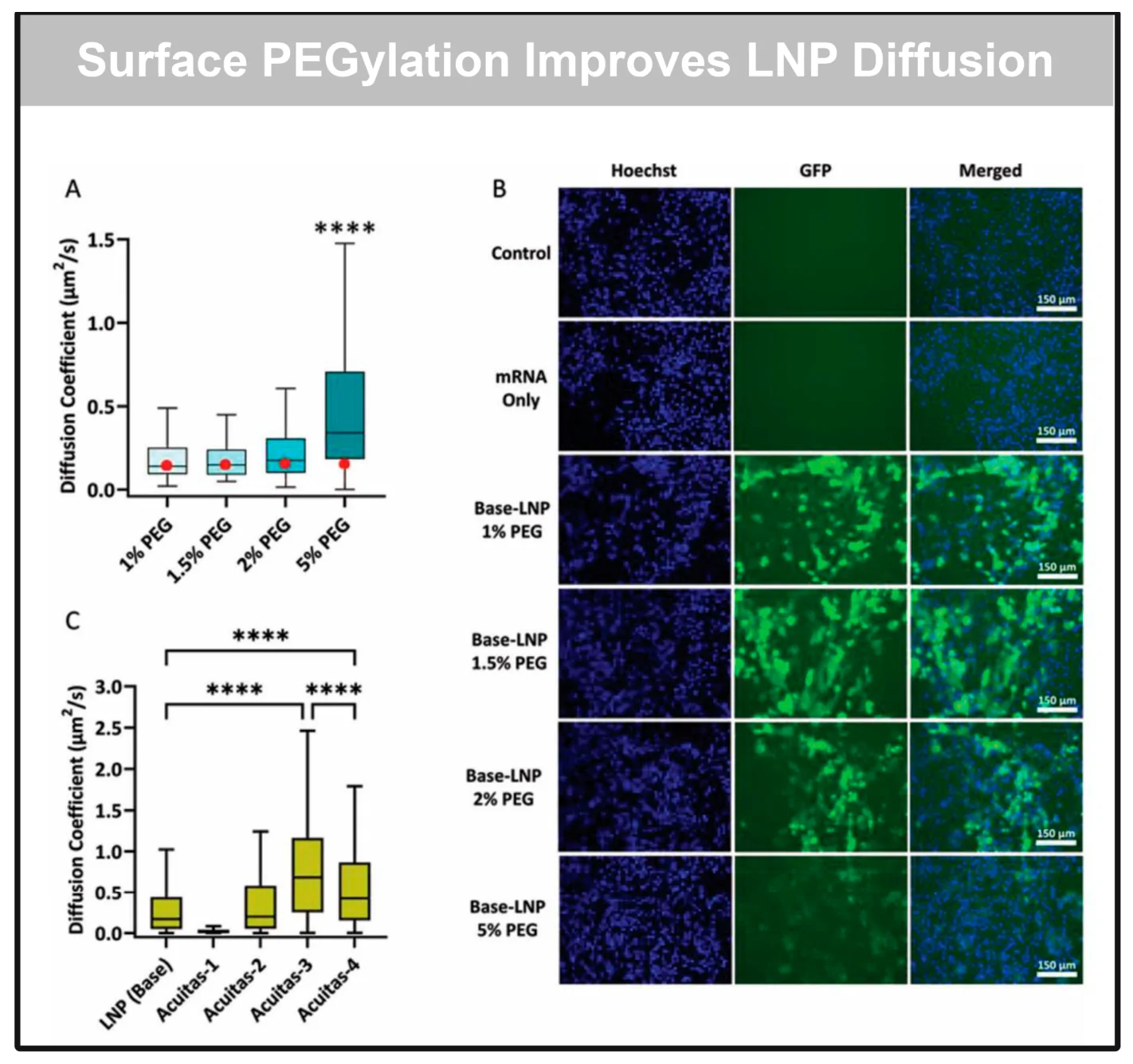
Surface PEGylation improves LNP diffusion [114]. (**A**) In mucin hydrogels, as PEG coverage increases, the diffusion coefficient D gradually increases, reaching statistical significance for 5% PEG. (**B**) Increasing PEG density had detrimental effects on transfection rates. (**C**) LNPs containing a mixture of PEG species had improved diffusion in CF mucus. Specifically, the median diffusivity of Acuitas-3 was 0.69 μm^2^/s, which is fourfold higher than the diffusion rate of the baseline LNP mRNA formulation (0.17 μm^2^/s) in CF mucus. **** means *p* < 0.0001. Reprint with permission from [114].

In an ex vivo porcine mucus model, Tafech et al. demonstrated that increasing the DMG-PEG content on LNPs from 1% to 5% gradually enhanced LNP mucus diffusion. However, this increase reduced functional GFP expression in primary cystic fibrosis bronchial epithelial cells. This indicates a trade-off between PEG density and transfection efficiency. However, the same study showed that this limitation could be mitigated by altering the type of PEG used. As shown in Figure 6, LNPs formulated with mixed PEG species (Acuitas-3, Acuitas-4) exhibited 2.5 to 4 times higher diffusion rates in CF mucus compared to single-species 1.5% PEG-LNPs, while maintaining GFP expression levels comparable to or exceeding those of high-PEG formulations [114]. Independently, Maniyamgama et al. varied the DSPE-PEG content in iLLN-2 from 0.5 mol% to 5 mol%, observing a gradual decline in in vivo nasal luciferase expression as PEG density increased. This was attributed to PEG-mediated surface shielding, which reduces cellular internalization [115]. Together, these studies indicate that PEG density is not a variable to be maximized indiscriminately; formulation optimization must balance mucus inertness with preserved cellular uptake according to delivery system requirements.

Parallel evidence suggests that free PEG in the LNP dispersion medium can enhance mucus penetration via a unique molecular weight-dependent mechanism. Wang et al. screened PEGs with molecular weights ranging from 400 to 10,000 Da as dispersing agents for DOTAP-based LNPs. Results showed that all molecular weights except PEG 10,000 improved transport in Transwell mucus models compared to PBS alone, with PEG 2000 showing the greatest enhancement. PEG 10,000 was proposed to entangle with mucin fibers through an interpenetrating polymer network effect, suggesting that excessively long PEG chains may impair rather than promote mucus transport [119].

In summary, while hydrophilic PEGylation effectively reduces mucin adhesion, it simultaneously suppresses serum protein adsorption and receptor-mediated endocytosis, thereby affecting cellular uptake efficiency. Resolving this conflict requires attention not only to PEG density but also to PEG chain length, molecular weight, and chemical type.

#### 3.3.4. Mobility Regulation

The mucus layer covering the mucosal surface is a complex fluid under dynamic renewal and shear forces, which requires the lipid bilayer on the surface of the delivery system to have corresponding adaptive properties. Therefore, the membrane fluidity directly determines whether nanoparticles are dynamically intercepted and restricted by the mucus microenvironment. Yu et al. prepared Lip 1-Lip 6 formulations that contained different phase transition temperature-assisted phospholipids, and compared their movement behavior in digestive tract mucus [120]. The results revealed that nanoparticles with high membrane fluidity could rapidly adjust the spatial distribution of their surface modification components upon contact with mucus to achieve instantaneous dissociation from mucin fibers, demonstrating significantly enhanced transmucosal permeation efficiency. Moreover, numerous studies have demonstrated that fluidity significantly influences nanoparticle cellular uptake, internalization pathways, and intracellular trafficking [121,122].

In studies related to LNPs, Maniyamgama et al. incorporated triolein into the iLLN formulation, enabling the formation of a liquid oil core within LNPs and thereby conferring enhanced particle fluidity. Using triolein-free iLLN-2 and a rigid-core variant prepared by substituting triolein with high-melting-point tristearin as controls [115]. The results demonstrated that the mucus penetration efficiency of triolein-containing iLLN-2 was significantly superior to both control groups. These findings indicate that increased fluidity facilitates particle deformation, thereby promoting mucus penetration.

Tam et al. systematically evaluated the transfection efficiency of six helper lipids (structural lipids), namely DSPC, DOPC, DOPE, DOPG, DOPS, and ESM. The results revealed that unsaturated oleoyl-based helper lipids (e.g., DOPS, DOPC) generally outperformed saturated stearoyl-based counterparts (DSPC) [116]. The unsaturated structural motif enhances LNP fluidity, which favors endosomal membrane fusion and consequently improves endosomal escape efficiency. However, the study identified a notable discrepancy between in vitro and in vivo outcomes: while DOPS exhibited the highest in vitro transfection efficiency, its in vivo transfection performance was relatively inferior in certain tissues. Conversely, ESM showed suboptimal in vitro activity but achieved the highest transfection efficiency in lung tissue in vivo. The authors proposed that these discrepancies may stem from differences in particle stability. Owing to its excessive fluidity, DOPS facilitates rapid membrane fusion but is prone to non-bilayer phase transitions, leading to structural instability. Upon prolonged exposure to complex in vivo biological environments, DOPS-containing LNPs may undergo premature dissociation or cargo release. In contrast, DOPC and ESM, with well-matched cross-sectional areas between their headgroups and acyl chains, readily form stable cylindrical bilayer structures, which confer a distinct advantage in in vivo settings.

#### 3.3.5. LNP Designs

In summary, when LNPs first deposit on the mucosal area, they need to traverse the mucus layer covering the epithelial surface to reach and interact with epithelial cells (or local antigen-presenting cells), followed by cellular uptake or transmembrane transport. Once internalized, they undergo intracellular trafficking; only if endosomal escape is triggered can functional expression within target cells be achieved or systemic absorption occur after transcellular transport. The four physicochemical properties, including particle size, surface charge, hydrophilicity/hydrophobicity (PEGylation), and membrane fluidity, significantly affect the mucosal penetration ability, internalization behavior, and lysosome escape capacity of LNPs.

When crossing the mucus layer, optimal LNPs possess four key characteristics. First, their hydrodynamic diameter should be smaller than the mesh size of the mucus network (100–500 nm for healthy mucus, 60–200 nm for CF mucus), ideally below 100 nm. Second, their surface zeta potential should be as close to neutral as possible, even when the apparent pKa of ionizable lipids is lower than the physiological pH of the mucosa. Third, the particle surface should be sufficiently hydrophilic due to PEGylation, preferably using medium-sized PEG molecules (e.g., 2000 Da). Fourth, the lipid bilayer on the particle surface should exhibit high membrane fluidity.

Then, LNPs contact epithelial cells. For effective local transfection of epithelial cells, nanoparticles should remain below 100 nm. In contrast, systemic immunization through antigen-presenting cells requires larger particles (200–500 nm), which are preferentially taken up by dendritic cells. Therefore, formulation design must make trade-offs based on application needs prior to this stage.

The optimal design for LNP cellular uptake/endocytosis conflicts with that for mucus penetration. While high PEG density (e.g., 5%) enhances mucus diffusion, it reduces uptake efficiency due to the shielding effect of PEG. Thus, formulations with low to moderate PEG density are more favorable for efficient cellular uptake and endocytosis. When both mucus penetration and cellular uptake need to be balanced, it is preferable to use mixtures of different PEG-lipid species (e.g., Acuitas-3, Acuitas-4). These LNPs maintain low overall PEG content without sacrificing uptake efficiency while achieving superior mucus diffusion rates compared to single-type high-density PEG formulations.

After internalization, LNPs enter intracellular trafficking. Their surface charge must change in response to environmental pH shifts, creating a responsive window for charge reversal during endosomal escape. During endosomal escape, the apparent pKa of the LNP should be within the range of endosomal acidification (pH 5.5–6.5) and slightly lower than the extracellular pH of mucosal tissues. For example, iLLN-2 (pKa 5.94) and DAS-LNP (pKa 6.30) meet this criterion. In contrast, LNPs containing only fixed cationic lipids (e.g., DOTMA) lacking pH responsiveness show significantly lower endosomal escape efficiency. Additionally, incorporating fusogenic helper lipids (e.g., DOPE-like structures) further enhances membrane destabilization and escape efficiency.

An ideal LNP should remain neutral, deformable, and moderately PEGylated during mucosal transit, while being capable of re-ionizing and inducing membrane fusion within the endosomal environment. Available evidence indicates that LNPs meeting both criteria can elicit significant mucosal IgA/IgG responses in murine intranasal administration models. Such formulations also achieve high nucleic acid accumulation and prolonged retention in bleomycin-induced murine pulmonary fibrosis models, with improved safety compared to positive controls. Conversely, if an LNP excels only in the mucus transit phase but lacks endosomal response design or possesses strong endosomal escape capability but poor mucus penetration, it will fail to achieve optimal therapeutic efficacy. However, the above conclusions are currently based primarily on specific animal models, in vitro studies, and limited cell line approaches. Their general applicability in human mucosal environments, primary epithelial cells, and other disease conditions remains to be validated.

## 4. Conclusions

Mucosal drug delivery offers a highly promising non-invasive route for the administration of large biomolecular drugs. With the development of lipid chemistry and nanotechnology, LNPs have made breakthroughs in the delivery of large molecules such as nucleic acids. An ideal LNP drug delivery system not only needs to protect fragile payloads like mRNA from the harsh pH and nuclease/protease environment at the mucosal surface but also must have the ability to rapidly penetrate the dense mucus layer and efficiently cross the epithelial cell barrier, ultimately releasing the drug intact at the site of action to exert its therapeutic effect.

Currently, although LNPs have significantly enhanced the in vivo stability of large biomolecules, their overall delivery efficiency is still limited by the complex physical and chemical barriers of the mucus and the absorption restrictions of epithelial cells. To further improve mucosal absorption efficiency, researchers have systematically regulated the physicochemical and surface properties of LNPs. The precise control of particle size, maintenance of near-electroneutrality, and construction of a high-density hydrophilic shell of the lipid membrane have significantly improved their diffusion and penetration behavior in the mucus to a certain extent. However, the properties of LNPs required to overcome the mucus barrier and the epithelial cell barrier are often contradictory. For instance, a highly hydrophilic and electrically neutral surface, which is beneficial for penetrating the mucus layer, severely hinders LNP electrostatic binding to the target cell membrane and subsequent endosomal escape. Therefore, future research on multifunctional LNPs urgently needs to develop intelligent delivery systems with environmental responsiveness, such as pH sensitivity and enzymatic cleavage, enabling dynamic changes in their physicochemical properties in a “spatiotemporal adaptive” manner when crossing different physiological barriers.

Additionally, due to the high diversity of LNP formulation components, the optimal parameters for mucosal crossing often vary among different research models, resulting in some optimization results lacking universality. Different immunization formulations, such as those targeting local mucosal sites and those aimed at systemic absorption, have different requirements. These must be considered differently based on the research objectives. LNPs designed for mucosal immunity need only penetrate the mucus and superficial epithelium to be captured by antigen-presenting cells for lymphatic activation. In contrast, non-immunogenic local gene therapy must achieve sustained parenchymal retention and functional delivery to non-epithelial targets such as alveolar macrophages and interstitial fibroblasts located outside the epithelial surface. This requirement becomes even more stringent when drugs are delivered via the mucosa for systemic effects.

Moreover, the mucosal microenvironment characteristics of different anatomical sites and even different patients are vastly different, requiring distinct LNP surface properties for optimal absorption. Therefore, it is necessary to closely integrate the pathological and physiological features of specific mucosal delivery sites and utilize multi-omics and artificial intelligence-assisted screening platforms to conduct “tailor-made” personalized regulation of LNPs.

Although LNP-based drugs such as MRT5005 and VX-522 have entered the early clinical trial stage based on mucosal delivery, converting them into successful clinical treatments remains a continuous challenge. MRT5005 was evaluated in phase I/II trials with single doses (8–24 mg) and multiple doses (up to 20 mg per week or 4 mg per day). However, the drug failed to consistently demonstrate efficacy in improving lung function, accompanied by significant systemic adverse events such as fever. Similarly, the clinical progress of VX-522 was suspended or halted recently in the multi-dose escalation phase due to tolerability issues. These setbacks highlight the strong physiological barrier of mucosal tissues and the local immune/inflammatory responses triggered by repeated administration of LNPs. Therefore, further conducting basic research to optimize the LNP formulation to adapt to mucosal delivery is a necessary step to overcome these delivery obstacles and safety limitations.

Taken together, translational gaps remain before mucosal LNP platforms can be reliably deployed in the clinic. Future work should prioritize scalable manufacturing processes for inhaled and oral LNP formulations, together with formulation stability during nebulization and gastric transit, as well as immunogenicity and toxicity evaluation. Meanwhile, standardized, reproducible in vitro and ex vivo mucus models with demonstrated predictive validity for human performance should be developed to bridge the persistent disconnect between preclinical potency and clinical translatability.

## Figures and Tables

**Table 1:** Classification and Representative Drugs of NADs.

Classification	Category	Representative Technology	Representative Varieties
Targeting Nucleic Acids	Regulating gene expression via base pairing	ASOs	Nusinersen [44]
		siRNAs	Patisiran [45]
		miRNAs [26]	-
		saRNAs [26]	-
		CRISPR/Cas9	Exa-cel [46]
Targeting Proteins	Bind to target proteins	aptamer drugs [47]	Pegaptanib (withdrawn) [48,49]
			Izervay
			AS1411 (in clinical development) [50,51]
Expressing proteins	Be translated into functional proteins	mRNA	BNT162b2 [52] mRNA-1273 [53]
			preventive vaccines [54,55,56,57,58]

## Data Availability

No new data were created or analyzed in this study. Data sharing is not applicable to this article.

## References

[1] Lu Z.-G., Shen J., Yang J., Wang J.-W., Zhao R.-C., Zhang T.-L., Guo J., Zhang X. (2023). Nucleic Acid Drug Vectors for Diagnosis and Treatment of Brain Diseases. Signal Transduct. Target. Ther..

[2] Ma P., He M., Lian H., Li J., Gao Y., Wu J., Men K., Men Y., Li C. (2024). Systemic and Local Administration of a Dual-siRNA Complex Efficiently Inhibits Tumor Growth and Bone Invasion in Oral Squamous Cell Carcinoma. Mol. Pharm..

[3] Zhang M., Wang Q., Wan K.-W., Ahmed W., Phoenix D.A., Zhang Z., Elrayess M.A., Elhissi A., Sun X. (2019). Liposome Mediated-CYP1A1 Gene Silencing Nanomedicine Prepared Using Lipid Film-Coated Proliposomes as a Potential Treatment Strategy of Lung Cancer. Int. J. Pharm..

[4] Tang X., Rao J., Yin S., Wei J., Xia C., Li M., Mei L., Zhang Z., He Q. (2019). PD-L1 Knockdown via Hybrid Micelle Promotes Paclitaxel Induced Cancer-Immunity Cycle for Melanoma Treatment. Eur. J. Pharm. Sci..

[5] Li J., Yao Y., Wang Y., Xu J., Zhao D., Liu M., Shi S., Lin Y. (2022). Modulation of the Crosstalk between Schwann Cells and Macrophages for Nerve Regeneration: A Therapeutic Strategy Based on a Multifunctional Tetrahedral Framework Nucleic Acids System. Adv. Mater..

[6] Yadav L., Kumar S., Srivastava S., Golmei P., Srivastava S., Kumar S., Sridhar S.B., Ravindra Babu M., Rahaman S.A. (2025). Aptamers: A Novel Class of Targeted Therapeutics. Biosensors and Aptamers: A New Era in Cancer Diagnosis and Treatment.

[7] Li M., Li M., Yang Y., Liu Y., Xie H., Yu Q., Tian L., Tang X., Ren K., Li J. (2020). Remodeling Tumor Immune Microenvironment via Targeted Blockade of PI3K-γ and CSF-1/CSF-1R Pathways in Tumor Associated Macrophages for Pancreatic Cancer Therapy. J. Control. Release.

[8] Wang S., Wang P., Song X., Ma X., Wei L., Zheng Y., Yu R., Zhang C. (2025). Site-Specific Dimerization of Interleukin-11 Alleviates Bleomycin-Induced Pulmonary Fibrosis in Mice. Eur. J. Pharm. Sci..

[9] Fu Q., Zhao Y., Yang Z., Yue Q., Xiao W., Chen Y., Yang Y., Guo L., Wu Y. (2019). Liposomes Actively Recognizing the Glucose Transporter GLUT1 and Integrin Avβ3 for Dual-Targeting of Glioma. Arch. Pharm..

[10] Zhang L., Zhao Y., Yue Q., Fu Q., Hai L., Guo L., Wang Q., Wu Y. (2018). Preparation and Characterization of GLUT1-Mediated Novel Brain Targeting Magnetic Nanoparticles. Lett. Drug Des. Discov..

[11] Shuai Z., Zheng Y., Jiang J., Yu R., Zhang C. (2021). Design BH3 Domain Fusion Protein as Targeting Pro-Apoptotic Self-Assembling Nanoparticles. Biomed. Pharmacother..

[12] Wang Y., Liu F., Wang Q., Xiang H., Jin H., Li H., Mao S. (2017). A Novel Immunoliposome Mediated by CD123 Antibody Targeting to Acute Myeloid Leukemia Cells. Int. J. Pharm..

[13] Weber J.S., Carlino M.S., Khattak A., Meniawy T., Ansstas G., Taylor M.H., Kim K.B., McKean M., Long G.V., Sullivan R.J. (2024). Individualised Neoantigen Therapy mRNA-4157 (V940) plus Pembrolizumab versus Pembrolizumab Monotherapy in Resected Melanoma (KEYNOTE-942): A Randomised, Phase 2b Study. Lancet.

[14] Wu H., Yu Y., Zhao Y., Liu W., Liu Z., Zhang G., Chen Z. (2023). A CH2CH3 Hinge Region Enhances the Cytotoxicity of Anti-CD5 CAR-T Cells Targeting T Cell Acute Lymphoblastic Leukemia. Int. Immunopharmacol..

[15] Yang T., Poenisch M., Khanal R., Hu Q., Dai Z., Li R., Song G., Yuan Q., Yao Q., Shen X. (2021). Therapeutic HNF4A mRNA Attenuates Liver Fibrosis in a Preclinical Model. J. Hepatol..

[16] Kamath D., Iwakuma T., Bossmann S.H. (2024). Therapeutic Potential of Combating Cancer by Restoring Wild-Type P53 through mRNA Nanodelivery. Nanomed. Nanotechnol. Biol. Med..

[17] Zhao Y., Yue P., Peng Y., Sun Y., Chen X., Zhao Z., Han B. (2023). Recent Advances in Drug Delivery Systems for Targeting Brain Tumors. Drug Deliv..

[18] Liang Y., Feng L., Zheng Y., Gao Y., Shi R., Zhang Z., Ying X., Zeng Y. (2024). Targeted Liposomal Co-Delivery Dopamine with 3-n-Butylphthalide for Effective Against Parkinson’s Disease in Mice Model. Int. J. Nanomed..

[19] Liao J., Ren X., Yang B., Li H., Zhang Y., Yin Z. (2019). Targeted Thrombolysis by Using C-RGD-Modified N,N,N-Trimethyl Chitosan Nanoparticles Loaded with Lumbrokinase. Drug Dev. Ind. Pharm..

[20] Li J., Yan Y., Zhang P., Ding J., Huang Y., Jin Y., Li L. (2022). A Cell-Laden Hydrogel as Prophylactic Vaccine and Anti-PD-L1 Amplifier against Autologous Tumors. J. Control. Release.

[21] Xia C., Li M., Ran G., Wang X., Lu Z., Li T., Tang X., Zhang Z., He Q. (2021). Redox-Responsive Nanoassembly Restrained Myeloid-Derived Suppressor Cells Recruitment through Autophagy-Involved Lactate Dehydrogenase A Silencing for Enhanced Cancer Immunochemotherapy. J. Control. Release.

[22] Pardi N., Hogan M.J., Porter F.W., Weissman D. (2018). mRNA Vaccines—A New Era in Vaccinology. Nat. Rev. Drug Discov..

[23] Tan L., Zheng T., Li M., Zhong X., Tang Y., Qin M., Sun X. (2020). Optimization of an mRNA Vaccine Assisted with Cyclodextrin-Polyethyleneimine Conjugates. Drug Deliv. Transl. Res..

[24] Zhang R., Tang L., Tian Y., Ji X., Hu Q., Zhou B., Ding Z., Xu H., Yang L. (2020). DP7-C-Modified Liposomes Enhance Immune Responses and the Antitumor Effect of a Neoantigen-Based mRNA Vaccine. J. Control. Release.

[25] Yi Y., Kim H.J., Zheng M., Mi P., Naito M., Kim B.S., Min H.S., Hayashi K., Perche F., Toh K. (2019). Glucose-Linked Sub-50-Nm Unimer Polyion Complex-Assembled Gold Nanoparticles for Targeted siRNA Delivery to Glucose Transporter 1-Overexpressing Breast Cancer Stem-like Cells. J. Control. Release.

[26] Naeem S., Zhang J., Zhang Y., Wang Y. (2025). Nucleic Acid Therapeutics: Past, Present, and Future. Mol. Ther. Nucleic Acids.

[27] Bangham A.D., Paoletti R., Kritchevsky D. (1963). Physical Structure and Behavior of Lipids and Lipid Enzymes. Advances in Lipid Research.

[28] Huang S., Zhang H., Zhang Y., Wang L., Zhang Z., Zhang L. (2023). Comparison of Two Methods for Tumour-Targeting Peptide Modification of Liposomes. Acta Pharmacol. Sin..

[29] Qiu Y., Yu Q., Liu Y., Tang J., Wang X., Lu Z., Xu Z., He Q. (2018). Dual Receptor Targeting Cell Penetrating Peptide Modified Liposome for Glioma and Breast Cancer Postoperative Recurrence Therapy. Pharm. Res..

[30] Yu Q., Qiu Y., Wang X., Tang J., Liu Y., Mei L., Li M., Yang M., Tang L., Gao H. (2018). Efficient siRNA Transfer to Knockdown a Placenta Specific lncRNA Using RGD-Modified Nano-Liposome: A New Preeclampsia-like Mouse Model. Int. J. Pharm..

[31] Zhou B., Li M., Xu X., Yang L., Ye M., Chen Y., Peng J., Xiao L., Wang L., Huang S. (2021). Integrin A2β1 Targeting DGEA-Modified Liposomal Doxorubicin Enhances Antitumor Efficacy against Breast Cancer. Mol. Pharm..

[32] Li R., Li Y., Zhang J., Liu Q., Wu T., Zhou J., Huang H., Tang Q., Huang C., Huang Y. (2020). Targeted Delivery of Celastrol to Renal Interstitial Myofibroblasts Using Fibronectin-Binding Liposomes Attenuates Renal Fibrosis and Reduces Systemic Toxicity. J. Control. Release.

[33] Costa C.P., Barreiro S., Moreira J.N., Silva R., Almeida H., Sousa Lobo J.M., Silva A.C. (2021). In Vitro Studies on Nasal Formulations of Nanostructured Lipid Carriers (NLC) and Solid Lipid Nanoparticles (SLN). Pharmaceuticals.

[34] Jung H.N., Lee S.-Y., Lee S., Youn H., Im H.-J. (2022). Lipid Nanoparticles for Delivery of RNA Therapeutics: Current Status and the Role of in Vivo Imaging. Theranostics.

[35] Ma Y., Li S., Lin X., Chen Y. (2024). A Perspective of Lipid Nanoparticles for RNA Delivery. Exploration.

[36] Li L., Luo M., Zhou L., Wang Y., Jiao Y., Wang C., Gong C., Cen X., Yao S. (2025). Glucocorticoid Pre-Administration Improves LNP-mRNA Mediated Protein Replacement and Genome Editing Therapies. Int. J. Pharm..

[37] Baral K.C., Choi K.Y. (2025). Barriers and Strategies for Oral Peptide and Protein Therapeutics Delivery: Update on Clinical Advances. Pharmaceutics.

[38] James H.A., O’Neill B.T., Nair K.S. (2017). Insulin Regulation of Proteostasis and Clinical Implications. Cell Metab..

[39] Sepasi T., Bani F., Rahbarghazi R., Ebrahimi-kalan A., Sadeghi M.R., Alamolhoda S.Z., Zarebkohan A., Ghadiri T., Gao H. (2022). Targeted Gene Delivery to the Brain Using CDX-Modified Chitosan Nanoparticles. BioImpacts BI.

[40] Zhang R., Tang L., Zhao B., Tian Y., Zhou B., Mu Y., Yang L. (2021). A Peptide-Based Small RNA Delivery System to Suppress Tumor Growth by Remodeling the Tumor Microenvironment. Mol. Pharm..

[41] Li M., Xie H., Liu Y., Xia C., Cun X., Long Y., Chen X., Deng M., Guo R., Zhang Z. (2019). Knockdown of Hypoxia-Inducible Factor-1 Alpha by Tumor Targeted Delivery of CRISPR/Cas9 System Suppressed the Metastasis of Pancreatic Cancer. J. Control. Release.

[42] Sun X., Setrerrahmane S., Li C., Hu J., Xu H. (2024). Nucleic Acid Drugs: Recent Progress and Future Perspectives. Signal Transduct. Target. Ther..

[43] Shi R., Zhu Y., Chen Y., Lin Y., Shi S. (2024). Advances in DNA Nanotechnology for Chronic Wound Management: Innovative Functional Nucleic Acid Nanostructures for Overcoming Key Challenges. J. Control. Release.

[44] Alhamadani F., Zhang K., Parikh R., Wu H., Rasmussen T.P., Bahal R., Zhong X., Manautou J.E. (2022). Adverse Drug Reactions and Toxicity of the Food and Drug Administration–Approved Antisense Oligonucleotide Drugs. Drug Metab. Dispos..

[45] Xiao B., Wang S., Pan Y., Zhi W., Gu C., Guo T., Zhai J., Li C., Chen Y.Q., Wang R. (2025). Development, Opportunities, and Challenges of siRNA Nucleic Acid Drugs. Mol. Ther. Nucleic Acids.

[46] Wu K., He M., Mao B., Xing Y., Wei S., Jiang D., Wang S., Alkuhali A.A., Guo J., Gan Z. (2024). Enhanced Delivery of CRISPR/Cas9 System Based on Biomimetic Nanoparticles for Hepatitis B Virus Therapy. J. Control. Release.

[47] Dhorde K.P., Roy I. (2026). Therapeutic Aptamers in Drug Discovery: Future Elements of the Pharmaceutical Arsenal?. Expert Opin. Drug Discov..

[48] Wang T., Chen C., Larcher L.M., Barrero R.A., Veedu R.N. (2019). Three Decades of Nucleic Acid Aptamer Technologies: Lessons Learned, Progress and Opportunities on Aptamer Development. Biotechnol. Adv..

[49] Ng E.W.M., Shima D.T., Calias P., Cunningham E.T., Guyer D.R., Adamis A.P. (2006). Pegaptanib, a Targeted Anti-VEGF Aptamer for Ocular Vascular Disease. Nat. Rev. Drug Discov..

[50] Rosenberg J.E., Bambury R.M., Van Allen E.M., Drabkin H.A., Lara P.N., Harzstark A.L., Wagle N., Figlin R.A., Smith G.W., Garraway L.A. (2014). A Phase II Trial of AS1411 (a Novel Nucleolin-Targeted DNA Aptamer) in Metastatic Renal Cell Carcinoma. Investig. New Drugs.

[51] Cheng Y., Zhao G., Zhang S., Nigim F., Zhou G., Yu Z., Song Y., Chen Y., Li Y. (2016). AS1411-Induced Growth Inhibition of Glioma Cells by Up-Regulation of P53 and Down-Regulation of Bcl-2 and Akt1 via Nucleolin. PLoS ONE.

[52] Lamb Y.N. (2021). BNT162b2 mRNA COVID-19 Vaccine: First Approval. Drugs.

[53] Verbeke R., Lentacker I., De Smedt S.C., Dewitte H. (2021). The Dawn of mRNA Vaccines: The COVID-19 Case. J. Control. Release.

[54] Aliprantis A.O., Shaw C.A., Griffin P., Farinola N., Railkar R.A., Cao X., Liu W., Sachs J.R., Swenson C.J., Lee H. (2021). A Phase 1, Randomized, Placebo-Controlled Study to Evaluate the Safety and Immunogenicity of an mRNA-Based RSV Prefusion F Protein Vaccine in Healthy Younger and Older Adults. Hum. Vaccines Immunother..

[55] Aldrich C., Leroux–Roels I., Huang K.B., Bica M.A., Loeliger E., Schoenborn-Kellenberger O., Walz L., Leroux-Roels G., von Sonnenburg F., Oostvogels L. (2021). Proof-of-Concept of a Low-Dose Unmodified mRNA-Based Rabies Vaccine Formulated with Lipid Nanoparticles in Human Volunteers: A Phase 1 Trial. Vaccine.

[56] Wei J., Hui A.-M. (2022). The Paradigm Shift in Treatment from COVID-19 to Oncology with mRNA Vaccines. Cancer Treat. Rev..

[57] Lorentzen C.L., Haanen J.B., Met Ö., Svane I.M. (2022). Clinical Advances and Ongoing Trials of mRNA Vaccines for Cancer Treatment. Lancet Oncol..

[58] Feldman R.A., Fuhr R., Smolenov I., Ribeiro A.M., Panther L., Watson M., Senn J.J., Smith M., Almarsson Ӧ., Pujar H.S. (2019). mRNA Vaccines against H10N8 and H7N9 Influenza Viruses of Pandemic Potential Are Immunogenic and Well Tolerated in Healthy Adults in Phase 1 Randomized Clinical Trials. Vaccine.

[59] Kulkarni J.A., Witzigmann D., Thomson S.B., Chen S., Leavitt B.R., Cullis P.R., van der Meel R. (2021). The Current Landscape of Nucleic Acid Therapeutics. Nat. Nanotechnol..

[60] Xiong L., Chen S., Li S., He D., Wang Y., Zhang Q., He Z., Li M., He Q. (2025). ATP-Responsive Tumor Targeted Lipid Nanoparticle for Enhanced siRNA Delivery and Improved Treatment Efficacy in Melanoma. J. Control. Release.

[61] Paunovska K., Da Silva Sanchez A.J., Sago C.D., Gan Z., Lokugamage M.P., Islam F.Z., Kalathoor S., Krupczak B.R., Dahlman J.E. (2019). Nanoparticles Containing Oxidized Cholesterol Deliver mRNA to the Liver Microenvironment at Clinically Relevant Doses. Adv. Mater..

[62] He C., Qin M., Sun X. (2020). Highly Pathogenic Coronaviruses: Thrusting Vaccine Development in the Spotlight. Acta Pharm. Sin. B.

[63] Arral M.L., Whitehead K.A. (2026). Design Principles of Lipid Nanoparticles for RNA Delivery. Nat. Rev. Bioeng..

[64] Aldosari B.N., Alfagih I.M., Almurshedi A.S. (2021). Lipid Nanoparticles as Delivery Systems for RNA-Based Vaccines. Pharmaceutics.

[65] Schoenmaker L., Witzigmann D., Kulkarni J.A., Verbeke R., Kersten G., Jiskoot W., Crommelin D.J.A. (2021). mRNA-Lipid Nanoparticle COVID-19 Vaccines: Structure and Stability. Int. J. Pharm..

[66] Xu Z., Seddon J.M., Beales P.A., Rappolt M., Tyler A.I.I. (2021). Breaking Isolation to Form New Networks: pH-Triggered Changes in Connectivity inside Lipid Nanoparticles. J. Am. Chem. Soc..

[67] Shepherd S.J., Han X., Mukalel A.J., El-Mayta R., Thatte A.S., Wu J., Padilla M.S., Alameh M.-G., Srikumar N., Lee D. (2023). Throughput-Scalable Manufacturing of SARS-CoV-2 mRNA Lipid Nanoparticle Vaccines. Proc. Natl. Acad. Sci. USA.

[68] Lopes C., Cristóvão J., Silvério V., Lino P.R., Fonte P. (2022). Microfluidic Production of mRNA-Loaded Lipid Nanoparticles for Vaccine Applications. Expert Opin. Drug Deliv..

[69] Zabaleta N., Unzu C., Weber N.D., Gonzalez-Aseguinolaza G. (2023). Gene Therapy for Liver Diseases—Progress and Challenges. Nat. Rev. Gastroenterol. Hepatol..

[70] Liu Y., Huang Y., He G., Guo C., Dong J., Wu L. (2024). Development of mRNA Lipid Nanoparticles: Targeting and Therapeutic Aspects. Int. J. Mol. Sci..

[71] Chen Y., Yang X., Li J., Luo H., Huang Q., Yang W., Lei T., Lui S., Gong Q., Li H. (2025). A Nasally Administrated Reactive Oxygen Species-Responsive Carrier-Free Gene Delivery Nanosystem for Alzheimer’s Disease Combination Therapy. J. Control. Release.

[72] Chen S., Tam Y.Y.C., Lin P.J.C., Sung M.M.H., Tam Y.K., Cullis P.R. (2016). Influence of Particle Size on the in Vivo Potency of Lipid Nanoparticle Formulations of siRNA. J. Control. Release.

[73] Cheng Q., Wei T., Farbiak L., Johnson L.T., Dilliard S.A., Siegwart D.J. (2020). Selective Organ Targeting (SORT) Nanoparticles for Tissue-Specific mRNA Delivery and CRISPR–Cas Gene Editing. Nat. Nanotechnol..

[74] Su L.-J., Wang N.-N., Luo R., Ji Z.-H., Gu H., Yang C., Xu M.-X., Zhang J., Chen Q., Yu M.-Z. (2026). Artificial Intelligence-Guided Design of LNPs for in Vivo Targeted mRNA Delivery via Analysis of the Spatial Conformation of Ionizable Lipids. Nat. Biomed. Eng..

[75] Xue L., Gong N., Shepherd S.J., Xiong X., Liao X., Han X., Zhao G., Song C., Huang X., Zhang H. (2022). Rational Design of Bisphosphonate Lipid-like Materials for mRNA Delivery to the Bone Microenvironment. J. Am. Chem. Soc..

[76] Breda L., Papp T.E., Triebwasser M.P., Yadegari A., Fedorky M.T., Tanaka N., Abdulmalik O., Pavani G., Wang Y., Grupp S.A. (2023). In Vivo Hematopoietic Stem Cell Modification by mRNA Delivery. Science.

[77] Chen J., Xu Y., Zhou M., Xu S., Varley A.J., Golubovic A., Lu R.X.Z., Wang K.C., Yeganeh M., Vosoughi D. (2023). Combinatorial Design of Ionizable Lipid Nanoparticles for Muscle-Selective mRNA Delivery with Minimized off-Target Effects. Proc. Natl. Acad. Sci. USA.

[78] Mahmoud D.E., Hosseini S.H., Rathore H.A., Alkilany A.M., Heise A., Elhissi A. (2025). Inhalable Nanotechnology-Based Drug Delivery Systems for the Treatment of Inflammatory Lung Diseases. Pharmaceutics.

[79] Gonsalves A., Menon J.U. (2024). Impact of Nebulization on the Physicochemical Properties of Polymer–Lipid Hybrid Nanoparticles for Pulmonary Drug Delivery. Int. J. Mol. Sci..

[80] Peng S., Wang W., Zhang R., Wu C., Pan X., Huang Z. (2024). Nano-Formulations for Pulmonary Delivery: Past, Present, and Future Perspectives. Pharmaceutics.

[81] Rowe S.M., Zuckerman J.B., Dorgan D., Lascano J., McCoy K., Jain M., Schechter M.S., Lommatzsch S., Indihar V., Lechtzin N. (2023). Inhaled mRNA Therapy for Treatment of Cystic Fibrosis: Interim Results of a Randomized, Double-blind, Placebo-controlled Phase 1/2 Clinical Study. J. Cyst. Fibros..

[82] Vertex Reports First Quarter 2026 Financial Results. https://news.vrtx.com/news-releases/news-release-details/vertex-reports-first-quarter-2026-financial-results.

[83] Bai X., Chen Q., Li F., Teng Y., Tang M., Huang J., Xu X., Zhang X.-Q. (2024). Optimized Inhaled LNP Formulation for Enhanced Treatment of Idiopathic Pulmonary Fibrosis via mRNA-Mediated Antibody Therapy. Nat. Commun..

[84] Xu Y., Ye X., Du Y., Yang W., Tong F., Li W., Huang Q., Chen Y., Li H., Gao H. (2025). Nose-to-Brain Delivery of Targeted Lipid Nanoparticles as Two-Pronged β-Amyloid Nanoscavenger for Alzheimer’s Disease Therapy. Acta Pharm. Sin. B.

[85] Tang Z., You X., Xiao Y., Chen W., Li Y., Huang X., Liu H., Xiao F., Liu C., Koo S. (2023). Inhaled mRNA Nanoparticles Dual-Targeting Cancer Cells and Macrophages in the Lung for Effective Transfection. Proc. Natl. Acad. Sci. USA.

[86] Sung J.C., Pulliam B.L., Edwards D.A. (2007). Nanoparticles for Drug Delivery to the Lungs. Trends Biotechnol..

[87] He X., Chen X., Wang H., Du G., Sun X. (2023). Recent Advances in Respiratory Immunization: A Focus on COVID-19 Vaccines. J. Control. Release.

[88] Jiang A.Y., Witten J., Raji I.O., Eweje F., MacIsaac C., Meng S., Oladimeji F.A., Hu Y., Manan R.S., Langer R. (2024). Combinatorial Development of Nebulized mRNA Delivery Formulations for the Lungs. Nat. Nanotechnol..

[89] Huang K., Liu Y., Miao H., Li Y., Fan Q., Zhang H., Zuo C., Zhu J., Zheng Q., Deng C. (2025). Nebulized Lipid Nanoparticles Based on Degradable Ionizable Glycerolipid for Potent Pulmonary mRNA Delivery. ACS Nano.

[90] Liu S., Wen Y., Shan X., Ma X., Yang C., Cheng X., Zhao Y., Li J., Mi S., Huo H. (2024). Charge-Assisted Stabilization of Lipid Nanoparticles Enables Inhaled mRNA Delivery for Mucosal Vaccination. Nat. Commun..

[91] Cheng X., Li Q., Zu H., Liu S., Li J., Wen Y., Yang C., Sun S., Lu H., Zhang Y. (2026). N-Acetylcysteine–Mediated Surface Remodeling of Inhaled mRNA Lipid Nanoparticles Enables Coordinated Mucosal and Systemic Antitumor Immunity. Adv. Mater..

[92] Huang J., Bai X., Stewart W., Xu X., Zhang X.-Q. (2025). Budesonide-Incorporated Inhalable Lipid Nanoparticles for antiTSLP Nanobody mRNA Delivery to Treat Steroid-Resistant Asthma. Nat. Commun..

[93] Hu B., Stewart W., Chen Q., Zhang C., Liu Z., Xu X., Zhang X.-Q. (2025). Modulating Tumor Collagen Fiber Alignment for Enhanced Lung Cancer Immunotherapy via Inhaled RNA. Nat. Commun..

[94] Vaca G.B., Meyer M., Cadete A., Hsiao C.J., Golding A., Jeon A., Jacquinet E., Azcue E., Guan C.M., Sanchez-Felix X. (2023). Intranasal mRNA-LNP Vaccination Protects Hamsters from SARS-CoV-2 Infection. Sci. Adv..

[95] Vu M.N., Pilapitiya D., Kelly A., Koutsakos M., Kent S.J., Juno J.A., Tan H.-X., Wheatley A.K. (2025). Deconvolution of Cargo Delivery and Immunogenicity Following Intranasal Delivery of mRNA Lipid Nanoparticle Vaccines. Mol. Ther. Nucleic Acids.

[96] Mehrdadi S. (2024). Lipid-Based Nanoparticles as Oral Drug Delivery Systems: Overcoming Poor Gastrointestinal Absorption and Enhancing Bioavailability of Peptide and Protein Therapeutics. Adv. Pharm. Bull..

[97] Zheng Y., Luo S., Xu M., He Q., Xie J., Wu J., Huang Y. (2024). Transepithelial Transport of Nanoparticles in Oral Drug Delivery: From the Perspective of Surface and Holistic Property Modulation. Acta Pharm. Sin. B.

[98] Ma Y., Dong S., Wu A., Jeong S.D., Lee A.S., Jiang W., Kim B.Y.S. (2026). Engineering Challenges and Translational Opportunities in Emerging Gene Delivery Platforms. Nat. Biomed. Eng..

[99] Wu L., Shan W., Zhang Z., Huang Y. (2018). Engineering Nanomaterials to Overcome the Mucosal Barrier by Modulating Surface Properties. Adv. Drug Deliv. Rev..

[100] Zhao Y., Liu C., Zhang X., Gu T., Wang Y., Zuo T., Liu S., Xu X., Meng H. (2026). Mechanistic and Strategic Insights into How Lipid Nanoparticles Interact with Membrane-Based Biological Barriers. ACS Nano.

[101] Suri K., Pfeifer L., Cvet D., Li A., McCoy M., Singh A., Amiji M.M. (2025). Oral Delivery of Stabilized Lipid Nanoparticles for Nucleic Acid Therapeutics. Drug Deliv. Transl. Res..

[102] Luo P.-K., Ho H.-M., Chiang M.-C., Chu L.-A., Chuang Y.-H., Lyu P.-C., Hu I.-C., Chang W.-A., Peng S.-Y., Jayakumar J. (2024). pH-Responsive β-Glucans-Complexed mRNA in LNPs as an Oral Vaccine for Enhancing Cancer Immunotherapy. Adv. Mater..

[103] Haque M.A., Shrestha A., Mattheolabakis G. (2025). Eudragit® S 100 Coating of Lipid Nanoparticles for Oral Delivery of RNA. Processes.

[104] Huang X., Liu C., Sharma S.N., You X., Chen S., Li Y., Liu H.-J., Liu B., Saiding Q., Chen W. (2025). Oral Delivery of Liquid mRNA Therapeutics by an Engineered Capsule for Treatment of Preclinical Intestinal Disease. Sci. Transl. Med..

[105] Cho C.W., Roh J., Lee S., Cho H.B., Lee J., So G., Na K., Kim H.J., Park K.-H. (2026). Orally Delivered, pH-Resistant Dual Coated Extracellular Vesicles Restore Intestinal Barrier Function and Suppress Colitis. Biomaterials.

[106] Gu X., Zhang Y., Gu F., Gao D., Deng W., Lu W., He J. (2026). Mucus-Penetrating Lipid Nanoparticles Overcome Lung Barriers for Pulmonary Co-Delivery of siGSDMD and pDNA-SAP in Idiopathic Pulmonary Fibrosis. Int. J. Pharm..

[107] Esih H., Mezgec K., Billmeier M., Malenšek Š., Benčina M., Grilc B., Vidmar S., Gašperlin M., Bele M., Zidarn M. (2024). Mucoadhesive Film for Oral Delivery of Vaccines for Protection of the Respiratory Tract. J. Control. Release.

[108] Van de Casteele I., Plovyt M., Stuchlíková M., Lanssens M., Verschueren B., Denon Q., Van der Meeren P., McCafferty S., Gitsels A., Cornillie P. (2026). Mucosal Administration of Lipid Nanoparticles Containing Self-Amplifying mRNA Induces Local Uptake and Expression in a Pig Model as a Potential Vaccination Platform against STIs. Drug Deliv. Transl. Res..

[109] Taketani Y., Usui T., Toyono T., Shima N., Yokoo S., Kimakura M., Yamagami S., Ohno S., Onodera R., Tahara K. (2016). Topical Use of Angiopoietin-like Protein 2 RNAi-Loaded Lipid Nanoparticles Suppresses Corneal Neovascularization. Mol. Ther.-Nucleic Acids.

[110] Sawaran Singh N.S., Gataa I.S., Saleh L.H., Ganesan S., Kavitha V., Maharana L., Sharma R., Latipova M., Madatova N., Jumanazarov D. (2026). Lipid Nanoparticle–Based mRNA Platforms for Mucosal HIV Vaccines: Formulation Advances, Immune Mechanisms, and Translational Pathways. Arch. Microbiol..

[111] Dawson M., Wirtz D., Hanes J. (2003). Enhanced Viscoelasticity of Human Cystic Fibrotic Sputum Correlates with Increasing Microheterogeneity in Particle Transport. J. Biol. Chem..

[112] Waheed S., Li Z., Zhang F., Chiarini A., Armato U., Wu J. (2022). Engineering Nano-Drug Biointerface to Overcome Biological Barriers toward Precision Drug Delivery. J. Nanobiotechnology.

[113] Schuster B.S., Suk J.S., Woodworth G.F., Hanes J. (2013). Nanoparticle Diffusion in Respiratory Mucus from Humans without Lung Disease. Biomaterials.

[114] Tafech B., Rokhforouz M., Leung J., Sung M.M., Lin P.J., Sin D.D., Lauster D., Block S., Quon B.S., Tam Y. (2024). Exploring Mechanisms of Lipid Nanoparticle-Mucus Interactions in Healthy and Cystic Fibrosis Conditions. Adv. Healthc. Mater..

[115] Maniyamgama N., Bae K.H., Chang Z.W., Lee J., Ang M.J.Y., Tan Y.J., Ng L.F.P., Renia L., White K.P., Yang Y.Y. (2025). Muco-Penetrating Lipid Nanoparticles Having a Liquid Core for Enhanced Intranasal mRNA Delivery. Adv. Sci..

[116] Tam A., Kulkarni J., An K., Li L., Dorscheid D., Singhera G., Bernatchez P., Reid G., Chan K., Witzigmann D. (2022). Lipid Nanoparticle Formulations for Optimal RNA-Based Topical Delivery to Murine Airways. Eur. J. Pharm. Sci..

[117] Laffleur F., Hintzen F., Shahnaz G., Rahmat D., Leithner K., Bernkop-Schnürch A. (2014). Development and In Vitro Evaluation of Slippery Nanoparticles for Enhanced Diffusion Through Native Mucus. Nanomedicine.

[118] Lai S.K., O’Hanlon D.E., Harrold S., Man S.T., Wang Y.-Y., Cone R., Hanes J. (2007). Rapid Transport of Large Polymeric Nanoparticles in Fresh Undiluted Human Mucus. Proc. Natl. Acad. Sci. USA.

[119] Wang X., Zhao Y., Huang X., Huang Y., Zhang Y., Xu C. (2025). Influence of Polyethylene Glycol on the Mucus Penetration and Stability of Lipid Nanoparticles in Cryopreservation and Lyophilization. RSC Adv..

[120] Yu M., Song W., Tian F., Dai Z., Zhu Q., Ahmad E., Guo S., Zhu C., Zhong H., Yuan Y. (2019). Temperature- and Rigidity-Mediated Rapid Transport of Lipid Nanovesicles in Hydrogels. Proc. Natl. Acad. Sci. USA.

[121] Sun J., Zhang L., Wang J., Feng Q., Liu D., Yin Q., Xu D., Wei Y., Ding B., Shi X. (2015). Tunable Rigidity of (Polymeric Core)–(Lipid Shell) Nanoparticles for Regulated Cellular Uptake. Adv. Mater..

[122] Bao C., Liu B., Li B., Chai J., Zhang L., Jiao L., Li D., Yu Z., Ren F., Shi X. (2020). Enhanced Transport of Shape and Rigidity-Tuned α-Lactalbumin Nanotubes across Intestinal Mucus and Cellular Barriers. Nano Lett..

